# Employment Instability and Childbirth over the Last 20 Years in Italy

**DOI:** 10.1007/s10680-023-09680-5

**Published:** 2023-10-12

**Authors:** Stefani Scherer, Elisa Brini

**Affiliations:** 1 University of Trento, Trento, Italy; 2 University of Oslo, Oslo, Norway

**Keywords:** Fertility, Employment instability, Insecurity, Italy, Partnership

## Abstract

Family formation is fostered by circumstances of plannability and economic and social stability. Conversely, as documented in previous literature, employment instability can hamper fertility decisions. Based on data from the Italian Labour Force Survey, this paper examines the association between employment-related instability and the likelihood of having a first or additional child from 2000 to 2020 in Italy, covering a period characterised by increasing labour market deregulation. Our results show that individual employment instability, such as temporary employment or unemployment, negatively influences the likelihood of having a first and second child, while the progression to higher parities is less affected by employment situations. Building upon previous research, we demonstrate how the negative association between fertility and employment instability has intensified over recent decades, especially for women. The large sample size also allowed for the examination of specific differences by educational levels and both partners’ employment situation. In contrast to traditional views about gender roles, the employment situation of one’s partner seems to matter less for women than for men.

**Supplementary Information:**

The online version contains supplementary material available at 10.1007/s10680-023-09680-5.

## Introduction

The increased diffusion of unstable employment situations due to widespread labour market deregulation has sparked considerable research interest in understanding their impact on the process of family formation (Blossfeld et al., 2006). This literature has documented that employment instability can hamper fertility decisions, although there are varying effects based on institutional contexts and between men and women (see Alderotti et al., 2021 for a recent review). In the southern European context, employment instability has significantly influenced families’ childbearing decisions, which some have attributed to less-developed state support for families and the scarcity of labour market dynamism (Barbieri et al., 2015). Previous literature on the consequences of employment instability on fertility decisions has focused primarily on the impacts of fixed-term contracts and unemployment (Di Nallo & Lipps, 2023; van Wijk et al., 2021, 2022).

Much of this literature has only looked at single historical time points and disregarded differences between social groups However, situations of employment instability might not only have become more common, but also changed in their quality and become more consequential for future employment situations (Alderotti et al., 2022; Barbieri et al., 2019b; Barbieri & Gioachin, 2022). Additionally, detailed findings beyond a differentiated impact of employment instability for men and women remain limited in existing studies (Buh, 2023), despite the fact that the consequences of insecure employment situations might substantially differ across social groups. Moreover, these analyses have often been limited to individuals without considering the presence and employment situation of a partner, who are critical, though often overlooked, actors in childbearing decisions (Di Nallo & Lipps, 2023; Doepke et al., 2022).

This paper contributes to the literature on employment instability by examining the case of Italy over a more extensive historical period, utilising 20 years of data from the Italian Labour Force Survey from 2000 to 2020. We investigated employment instabilities by focusing on fixed-term contracts and unemployment compared to permanent employment contracts. Our analysis also takes into consideration self-employment and inactivity.

Italy presents a noteworthy case not only for its remarkably low fertility levels—a total fertility rate (TFR) of 1.24 compared to an average European TFR of 1.53 in 2020 (OECD, 2023)—but also because it underwent a process of labour market deregulation (see below), which likely increased overall employment instability and its social consequences. Therefore, the primary objective of our analysis was to document the effects of employment instability over a more extensive period. While doing so, we also accounted for other sources of instability, including occupational positions, salaries, and unemployment levels. We then examined the differences in these factors across genders and educational levels. Finally, we extended the scope beyond the individual level to also include the partner’s situation for those in a couple, studying the effects of employment-related insecurities on both partners. Diverging from much of the existing research that has predominantly focused on the transition to parenthood, we examined first-, second-, and high-order births, recognising that the dynamics underlying the decision to have one’s first child might differ from having an additional child (Kreyenfeld & Andersson, 2014).

## Background

### Employment Instability and Fertility

Procreation in many developed countries is often tied to stability in living conditions, with individuals being motivated to have a child when they can guarantee to provide ample economic support (Kohler et al., 2022; Oppenheimer, 1994; Vignoli et al., 2020a). There is extensive empirical evidence that fertility decisions are heavily influenced by past and current employment-related instability, often measured in terms of temporary employment contracts, unemployment, and other precarious economic situations (Andersson et al., 2014; Kreyenfeld et al., 2012; van Wijk et al., 2022; Vignoli et al., 2020a, 2020b). Previous studies have suggested that instability hampers fertility not only in terms of timing but also in terms of quantum (Alderotti et al., 2022; Ciganda, 2015; Clark & Lepinteur, 2022; Pailhé & Solaz, 2012).

However, the exact consequences of instability are highly dependent on the specific type of instability, as well as national contexts (Ayllón, 2019; Comolli & Vignoli, 2021; Kristensen & Lappegård, 2022). A recent meta-analysis by Alderotti et al. (2021) suggested that the impact of temporary employment on fertility has grown stronger over time. While some studies have found that temporary employment has no effect on parenthood in some countries (de Lange et al., 2014; Wolbers, 2007), there appears to be a pronounced correlation in the southern European context (Barbieri & Bozzon, 2016; Barbieri et al., 2015).

The various aspects of instability can overlap to some extent, as temporary employment not only lacks stability and comes with higher risks of unemployment but also tends to involve lower salaries, poorer working conditions (Barbieri & Cutuli, 2010; OECD, 2014), and more limited economic resources (e.g. van Wijk et al., 2021, 2022). For instance, van Wijk et al. (2021) suggested that, in the Netherlands, the consequences of temporary employment often are attributable to lower income.

Much of the existing literature has disregarded long-term trends by focusing on specific temporal moments. However, there are reasons to believe that the impact of employment instability on fertility decisions is not stable over time. We provide this reasoning by examining the specific case of Italy.

### The Italian Context and the Increasing Relevance of Employment Instability

Like many other Western European countries, the Italian labour market witnessed extensive deregulation over the last two decades. Labour market deregulation was initiated in 1997 (Riforma Treu, Law No. 196/1997) with additional changes in 2003 (Riforma Biagi, Law No. 30/2003) when regulations for temporary employment were radically reduced, yielding significant increases in temporary and perpetuated temporary employment without substantially affecting unemployment levels, which have remained relatively high, particularly among labour market entrants. Since then, there has been a marked proliferation of non-permanent contracts, whose nature has become increasingly unstable (Barbieri & Scherer, 2009; Hipp et al., 2015).^1^

These normative changes in the nation’s employment fostered a process of labour market dualisation (Barbieri & Cutuli, 2016; Emmenegger et al., 2012), which establishes boundaries between well-sheltered ‘insider’ and un-protected ‘outsider’ workers employed in the secondary labour market sector, implying the rising risk of remaining entrapped in insecure and underpaid jobs (Blanchard & Landier, 2002). As in other contexts, labour market deregulation in Italy mainly occurred ‘at the margins’—leaving the regulation of core workers rather untouched (Barbieri, 2009; Bentolila et al., 2021; Rubery, 2015). But more than in other contexts, the dualisation of the Italian labour market followed a cohort divide, increasingly concentrating forms of unstable employment on young people in their reproductive age (Barbieri & Scherer, 2009; Cutuli & Guetto, 2013).

As the literature has shown, labour market deregulation and dualisation have been significantly consequential for fertility decisions through the increased diffusion of unstable employment situations, such as temporary employment and repeated unemployment, which hamper fertility. Further, the increased dualisation might have made these employment situations increasingly less compatible with family formation. This brings us to our first hypothesis: *we expect that the increasing levels employment instability and dualisation brought about by deregulation led to increasingly negative impacts of non-permanent employment and unemployment for fertility over time (H1)*.

The labour market situation in Italy, coupled with other structural conditions, can intensify the detrimental impact of employment instability on fertility decisions. Aligned with what has been labelled the ‘Southern Model of Welfare’ (Ferrera, 1996), Italy provides limited public support to families regarding transfers and especially services, making children a rather costly investment. Moreover, the division of partnership roles has generally followed traditional patterns, with women’s employment participation still being relatively low (Barbieri et al., 2019a), while housework largely remains women’s work (Dotti Sani & Scherer, 2018; Matteazzi & Scherer, 2021). Low levels of welfare support for families and gender imbalances in housework (Goldscheider et al., 2015) support the expectations of considerable differences in the way employment situations of men and women influence fertility decisions, but not necessarily in the ‘traditional’ way. This brings us to the point that employment instability may influence different social groups in disparate ways.

### Gendered Consequences of Employment Instability and the Role of Partners

There are well-documented differences between men and women in terms of the impact of employment situations on each. Oppenheimer’s Uncertainty Thesis (1994) referred to men, stating that employment insecurity hampers male ‘breadwinning’ capacities and, thus, negatively affects family formation. Conversely, women dispose of alternative roles and may invest in care work to compensate for lacking employment success, which has varying effects on fertility decisions (Friedman et al., 1994). While unemployment has been shown to have a positive association with motherhood (Schmitt, 2012a) and a negative relationship with fertility in men (Kristensen & Lappegård, 2022; Pailhé & Solaz, 2012), studies examining the effects of temporary employment on parenthood for men and women have yielded mixed results. Some have found temporary work to correlate with postponed parenthood for both men and women, such as in Finland (Sutela, 2012), France (Pailhé & Solaz, 2012), Sweden (Lundström & Andersson, 2012), and Italy (Vignoli et al., 2012, 2020b). However, other studies have reported negative effects on women but not on men, such as in Australia (Laß, 2020), Germany (Auer & Danzer, 2021; Laß, 2020; Schmitt, 2012b), Spain, and Italy (Barbieri et al., 2015).

Over time, the association between employment instability and fertility for men and women may have changed, as the empirical regularities observed when Oppenheimer developed her thesis no longer hold today. These include women’s increased labour market participation, improved career prospects, and their educational ‘primacy’, as well as evolving societal attitudes towards gender equity. Moreover, women’s employment plays a more vital role in their and their family’s well-being, which extends beyond times of economic crisis (Vitali & Arpino, 2016), even within traditionalist contexts like Italy (Brini et al., 2021).

When deciding to start a family, it is likely that women today not only consider the economic stability and employment prospects of their partner’s employment but also, more than before, of themselves. As women have gained greater economic independence, the traditional association between men’s stable employment and family formation may have become less prominent. Since having a child is usually a couple’s mutual decision, both partners’ situations will likely influence it (Nitsche et al., 2018).

However, only a few studies have explicitly considered the employment situations of partners in tandem (Latshaw & Yucel, 2022). Among those, some have suggested that men’s, rather than women’s, employment situations might be more relevant for family decisions (Busetta et al., 2019) because women can often count on a male ‘breadwinner’ (Vignoli et al., 2012). Nevertheless, although men’s earnings and career chances are still higher than women’s (Matteazzi & Scherer, 2021), and male-dominated households are still quite common (Kowalewska & Vitali, 2021), the position of women in the household has significantly increased in relevance. Among younger cohorts, most women remain employed after childbirth (Musick et al., 2020). Women’s employment and income have also become impactful on family budgets, combined with an erosion of the breadwinning capacity of men, and women often do not need to rely on a partner to provide a family income (Vignoli et al., 2012). Therefore, their employment situation may have become more relevant for family planning decisions. Partners also tend to be increasingly similar across cohorts regarding social positions, education, and employment situations (Corti & Scherer, 2021; Grotti & Scherer, 2014). Thus, even in more traditional contexts like Italy, women’s employment situation has become increasingly relevant**,** even though the presence of an employed male partner often remains paramount. Many studies have documented the (increasingly) negative effects of employment insecurities on women’s fertility decisions (Alderotti et al., 2021; Schmitt, 2012b), although there has been little evidence for Italy thus far.

From these reflections, we derive two hypotheses, one about gender differences in general and another about the relevance of the partner’s situations within couples. First, *we expect that women’s employment instability has, in general, become more influential in determining fertility decisions, leading to reduced gender differences in the relationship between temporary employment and fertility (H2).* Second, *we expect that, in general, both partners’ employment situations are relevant, but women’s employment stability within the couple has become more decisive for family formation (H3)*.

Partnership formation is a stratified process (Schwartz, 2013), and not all individuals are equally desirable partners; just like in any other market (Oppenheimer, 1988), desirability depends on preferences and structural constraints (Corti & Scherer, 2021). Along with a general postponement in union formation, there is an increasing rate of singlehood (Bellani et al., 2017), mostly with low-educated men who may be deemed less ‘attractive’ to potential partners. Similarly, an advantageous labour market position is an essential characteristic for success in the marriage market, especially for men (Corti & Scherer, 2021). Thus, the correlation between lower fertility and unstable employment situations might be due to lacking a partner. Therefore, we will consider a partner’s presence before turning to their characteristics.

### Heterogeneity by Education and Parity

Gender differences are not the only heterogeneities to be expected in terms of employment instability and fertility; there may also be different consequences of employment instability for fertility according to a person’s human capital (and the quality of their occupation). The reasons for this comprise arguments both about the employment and economic consequences of unstable employment situations as well as the expectations of the parental role, which differ across social groups characterises by different educational levels.

On the one hand, employment instability is often more persistent for less educated individuals (Wolbers, 2000), while higher-educated individuals are more likely to use fixed-term contracts as stepping stones for gainful employment (Scherer, 2004). On the other hand, lower-educated women have weaker employment attachment (Dotti Sani & Scherer, 2018), which contributes to the alternative roles mentioned above. Highly educated women may be more inclined than those with lower levels of education to prioritise their career advancement over starting a family until they have attained stable employment (Pailhé & Solaz, 2012). At the same time, expectations related to parenthood and parental roles vary by educational level. Families usually pursue reproducing their social status or increasing it within their children (Breen & Goldthorpe, 1997; Mare, 2014), which implies high economic investments for highly educated parents. Overall, *we might expect that employment instability is more diametral to having children for highly educated individuals (H4)*, especially for first births in women as parenthood is postponed.

While much of the literature has concentrated on first births, the relevance of employment instability for fertility outcomes might differ depending on whether individuals are considering becoming a parent for the first time or having an additional child (Kreyenfeld & Andersson, 2014). The transition to parenthood signifies a substantial transformation in parents’ financial circumstances and work-life equilibrium (Musick et al., 2020), making job stability crucial as it signals to prospective parents their ability to maintain a reliable income or career while managing their childcare responsibilities. When contemplating the prospect of having another child, people may have already achieved a certain level of career stability or negotiated their roles and responsibilities within the household. It follows that employment stability may be less influential on the decision to have subsequent children. Research by Lopes (2020) in Portugal supports this idea, showing that job security has a more pronounced effect on fertility choices for the first child compared to decisions regarding subsequent births. In line with this, *we separately analyse the transitions to first, second, and higher-order births and expect the transition to parenthood to be more sensitive to employment instability compared to the transition to subsequent children (H5).*

## Data and Methods

### Data and Variables

We conducted our empirical analysis using the Labour Force Survey (LFS) for Italy from 2000 to 2020 (Eurostat, 2021; GESIS, 2023). Although cross-sectional in nature, the LFS data incorporates a longitudinal component of information collected one year before the interview on key dimensions relevant to our study. These data are not ideal, as explained below, but include several fundamental advantages for our study. First, the large samples allow for a detailed analysis of specific social groups, primarily through the intersection of gender and education in this case. Second, they include the employment situation of both partners (if they live in the same household). Finally, they cover a relatively long time, allowing us to consider the effects of the deregulation processes in the Italian labour market. Therefore, the survey data allow for an extension of existing research by incorporating relevant changes over time and systematically considering the heterogeneity of individuals and the characteristics of their partners. Although the data do not facilitate the identification of causal effects, for readability, we use terms like ‘effect’ to indicate associations without necessarily implying a causal relation.

The initial sample included persons aged 15 to 49 (results are robust to alternative age bounds), not retired or permanently disabled, not studying or in military service, and comprised 3,551,953 individuals (1,758,968 men and 1,792,985 women), reduced to 3,550,795 once all missing values were excluded. Descriptive statistics are available in Table 1 in ‘Appendix’.

Fertility was reconstructed through the presence and age of children in the household. More precisely, we identified a birth through the presence of a child below the age of one. This ‘own-child’ method is not ideal, but it has been well-used in previous studies to approximate fertility for the considered age range (Brini, 2020; Krapf & Kreyenfeld, 2015). However, especially for men, relying on children present in the household can underestimate fertility, as children tend to live with their mothers when their parents do not share the same household.^2^

Parity was distinguished by the presence of older children.^3^ We distinguished between first, second, and all other higher-order births. In detail, we observed 57,154 first births (subjects at risk: 1,572,165), 60,117 second births (at risk: 1,006,876), and 21,777 higher-order births (at risk: 1,089,025). About 4% of the individuals in our sample experienced a birth in the observation window (4% first birth, 6% second births, and 2% third births). The share was slightly higher for women (especially for first births), and the time trend was in line with data on period fertility.

Employment situation was considered a lagged measure based on an individual’s main status in the year preceding the interview. This measure is also not ideal as some children might have been conceived before this date, but it is the only information available; however, the Italian labour market is not very fluid (Barbieri & Cutuli, 2016), so the connected errors are arguably limited.^4^

The employment situation measure distinguishes between permanent employment,^5^ self-employment, temporary employment,^6^ unemployment, and inactivity (including care work and leaves). We considered unstable employment situations as temporary/fixed-term contracts and unemployment, which allowed us to analyse the implications of several forms of employment instability. We compared these metrics to the fertility behaviours of people with permanent employment contracts and extended the analysis to inactive and self-employed individuals. Self-employment has been considered a potential solution for women seeking a better work-family balance, especially in countries with limited public support for work-life reconciliation (Wellington, 2006), and has been associated with higher fertility in some countries (e.g. Coppola & Di Cesare, 2008 for Spain). In Italy, where self-employment has been traditionally associated with men, female self-employment primarily consists of poorly qualified and low-skilled occupations (Barbieri & Bison, 2004) and has not been linked to higher fertility (Coppola & Di Cesare, 2008; Del Boca et al., 2005). Self-employment might be subject to increasing instability, which is why its inclusion is relevant.

**Fig. 1:**
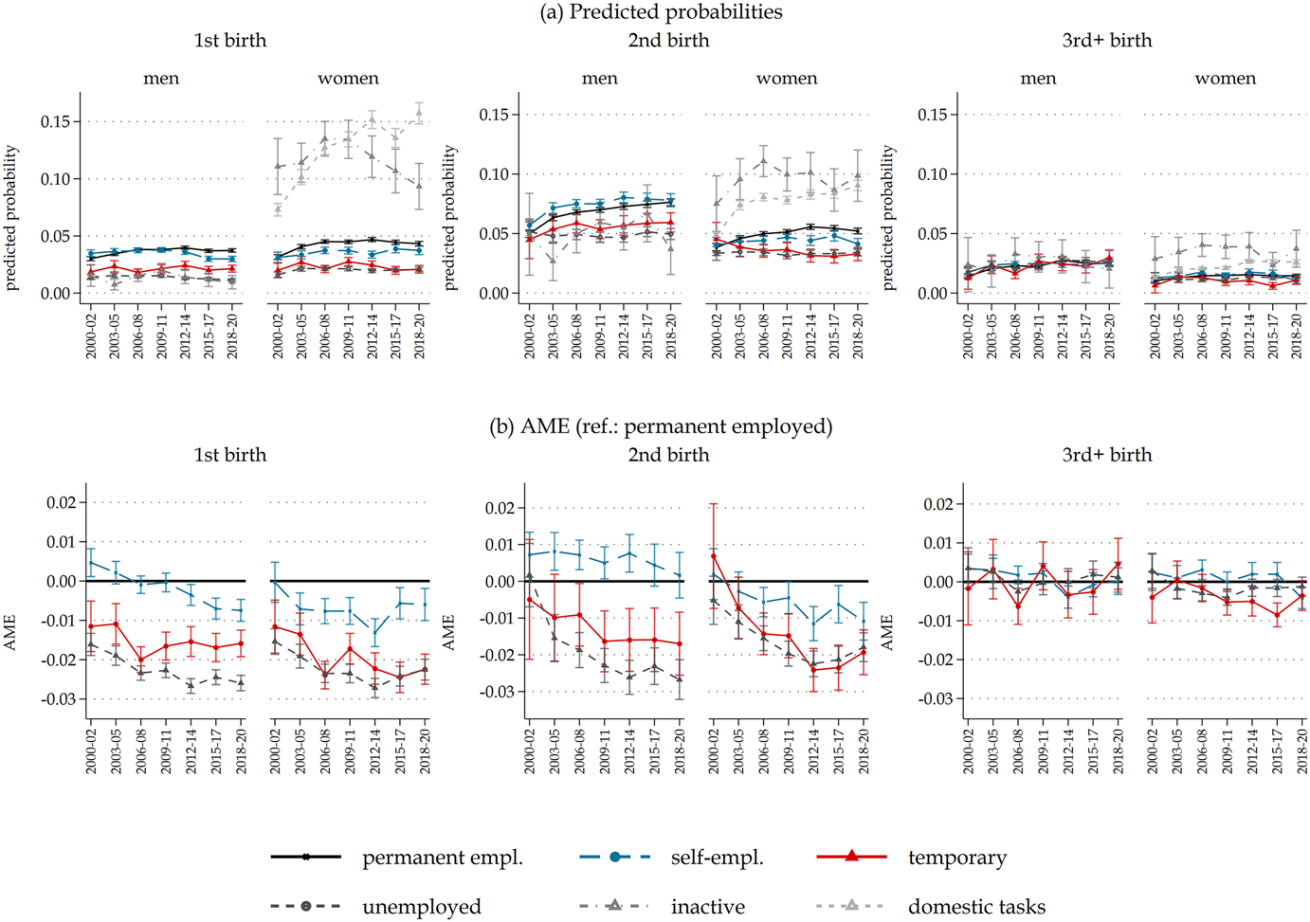
Childbirth and employment situation over time. * Source*: LFS, Italy 2000–2020. *Note*: Predicted probabilities (**a**) and average marginal effects (**b**) from logistic regression models examining first, second, and higher childbirth as a function of employment situation (1-year lagged) by sex and period. The results are based on nonparametric time trends and adjusted for age, age-squared, and level of education, and include 95% confidence intervals. Panel (b) omits the estimates for those not in the labour force (inactive), which are available in ‘Appendix Fig. 5’

In line with previous research, time trends in employment status (‘Appendix, Fig. 1’) showed an increase in unemployment (from 12% to 19%) and temporary employment (5% to 12% for men and 5% to 10% for women) for the period in consideration, on our sample. Self-employment remained stable for women at around 9% and slightly declined for men by five percentage points (p.p.) to 17%. There was also a net decline of inactivity among women from 33% to 20%.

To address heterogeneous effects beyond gender, we also considered educational level. Based on the highest educational attainment, we distinguished between individuals with low (below upper secondary), middle (upper secondary), or high (tertiary level) levels of education. The partner’s employment situation, as with the respondent, refers to the year preceding the interview and distinguishes across the same employment statuses. Overall, approximately 60% of the people included in our sample had a partner.

In the supplementary model specification, we included a control for the individual’s and partner’s net salary, social class, and regional levels of youth unemployment. Salary refers to monthly take-home pay from the main job and is available in deciles from 2009 limited to employees, resulting in a shorter time period and a restricted sample of dependent employees for our analyses. Social class was measured according to the literature on occupational social class, applying an aggregated version of the European Socio-Economic Group (ESeG) classification (Rose & Harrison, 2007), which distinguishes between upper, middle, and lower class^7^ based on International Standard Classification of Occupations (ISCO) codes. These measures were only available at the time of the interview. Regional levels of youth unemployment were measured one year before the observation as the share of people aged 15–39 who were unemployed. Its inclusion, at least partially, incorporates the effects of economic business cycles.

### Analytical Strategy

Our results are based on logistic regression models of first, second, or higher births on 1-year lagged employment situations with robust standard errors. Additional checks were also conducted using Poisson regressions applied to the binary outcomes and complementary log–log models to assess possible downward biases of the logistic regressions due to births being rare events (King & Zeng, 2001; Zou, 2004). These alternative specifications did not yield significant differences (Online Resources, Table S1).

To examine changes in the association between employment instability and fertility over time, we introduced an interaction term with a nonparametric specification of time, distinguishing three-year periods. Various time specifications were tested, as described in the ‘Robustness Check’ section.

Our results distinguish between men and women. Our preferred model specification included controls for age, age-squared, and education, although we tested the association between employment instability and fertility without any controls, as well as with additional controls, as mentioned above. After reporting the results for all individuals, we limited the analysis to those living in a partnership and controlled for partner’s employment status.

To test the extent to which the effects of employment instability are mediated through economic instability, we controlled for salaries (measured in deciles) and respondents’ occupational positions (Online Resources, Tables S2–S3) in our supplementary analyses. We also repeated this for couples (Online Resources, Table S4). Since salaries were available only for dependent employees, this limited our analysis to a comparison of temporary and permanent contracts. Additionally, salaries and occupational positions were collected only with reference to the same period as the measurement of childbirth and available for periods after 2009; therefore, we utilised this information only for our supplementary models.^8^ It is worth noting that the substantive results persisted when these variables were included in the models. We also included a quadratic specification of regional unemployment levels as a potential confounder (Online Resources, Tables S2–S3).

**Fig. 2:**
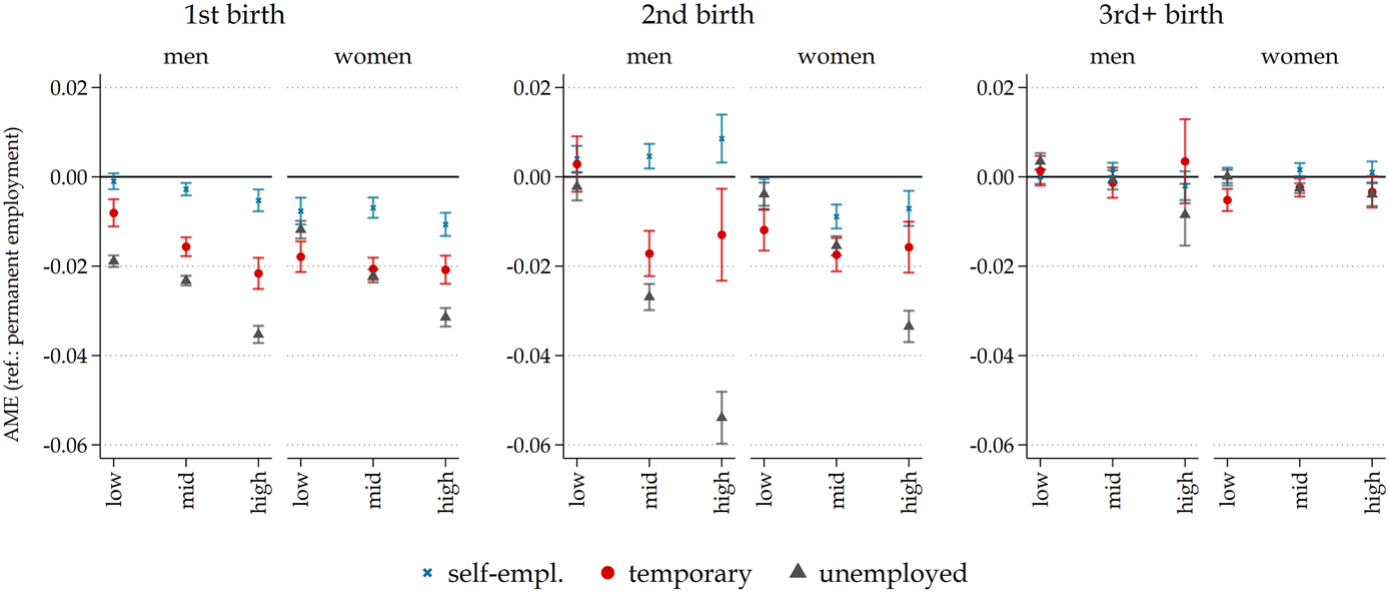
Moderation by education. *Source*: LFS, Italy 2000–2022. *Note*: Average marginal effects (AME) from logistic regression models examining first, second, and higher childbirths as a function of employment situation (1-year lagged), by sex and educational level, controlling for age, age-squared, and period, with 95% confidence intervals. ‘Low’ refers to below upper secondary, ‘mid’ to upper secondary, and ‘high’- to tertiary-level education

To test whether employment instability affects the fertility of men and women with different levels of education, we focused on the active labour force (i.e. excluding individuals who were inactive in the year prior to the interview) (Fig. 2). These models also included controls for age, age-squared, and nonparametric specifications of the period. We conducted further supplementary analyses by considering the individual’s social class and salary, which did not significantly alter the findings (Online Resources, Figure S1).

**Fig. 3:**
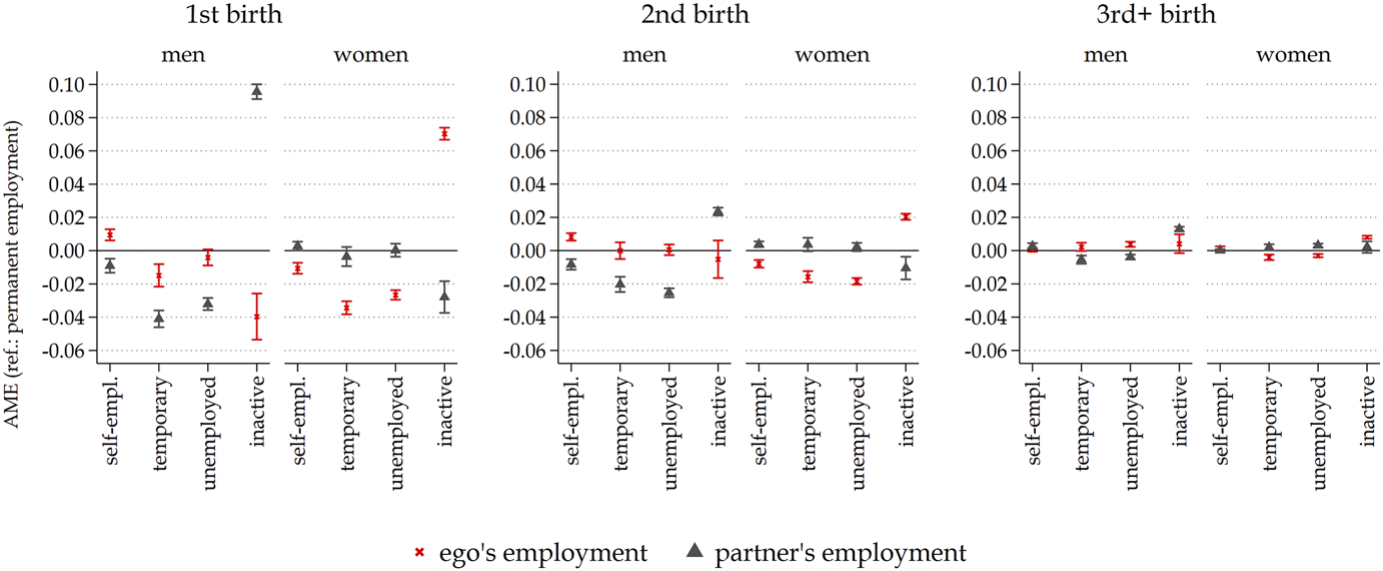
Own and partner’s employment situation. *Source*: LFS, Italy 2000–2020. *Note*: Average marginal effects (AME) from logistic regression models examining first, second, and higher childbirths as a function of one’s own employment situation (1-year lagged) and partner’s employment situation (1-year lagged), by sex, controlling for age, age-squared, level of education and period, with 95% confidence intervals

Finally, to investigate whether and how the employment situation of both partners influences fertility (Fig. 3), we limited our sample to individuals who cohabited with a heterosexual partner and considered the employment status of the respondent and partner together. Again, these results are reported separately for men and women. Analyses on how the association between employment and fertility differs between people with and without a partner and the results, including controls for household income and both partners’ social class, are given in ‘Appendix Fig. 6’ and Figure S2 in the Online Resources, respectively.

As our interest was in associations rather than distributions, all results are based on unweighted analyses. However, the results did not differ substantively with and without weights. Results from the logistic regression are presented both in terms of the predicted probability of experiencing a first, second, or subsequent childbirth based on the employment situation for men and women or in terms of average marginal effects (AME) with permanently employed as the reference category.

## Results

### Consequences of Employment Instability

Our results confirm the importance of one’s employment situation for childbirth and the differences between men and women. Panel A of Fig. 1 illustrates the predicted probabilities for men and women experiencing childbirth, distinguishing first, second, and third or higher births based on the previous year’s employment situation. We found that unemployment and temporary employment situations  lower probabilities of experiencing childbirth. Inactivity the previous year did promote childbirth for women but not for men. For women, the predicted probabilities of childbirth distinguished ‘inactivity’ from ‘inactivity due to domestic tasks’. Both states demonstrated notably higher chances of birth, especially for first births, and those with ‘domestic tasks’ even showed increasing probabilities of childbirth over time. Most women who reported being inactive identified their inactivity resulting from domestic tasks (about 93% of all inactive women), which was virtually absent among men (less than 0.3% overall) which is why the two inactivity categories are aggregated. Given that our primary interest is in the effects of employment situations, Panel B of Fig. 1 shows the active population only and reports the AME in reference to dependent employment, the effects of which proved to be relatively stable over time.

Unstable employment situations (i.e. temporary contracts and unemployment) and, to a lesser extent, self-employment for women yielded lower chances of childbirth. Differences in childbirth for these individuals compared to those with permanent employment were substantial, with temporary employment lowering the chance for a first birth by more than two percentage points for a woman in 2018–2020 compared to those with a permanent job (on an incidence of 4.31% of first births and 5.24% of second births in the related analytical sample). Regarding first and second births, the results were relatively similar. The picture regarding third births is slightly different, and the effects were much smaller. For men, the employment situation seems to have had no relevant influence.

Differentials between employment statuses widened over the observed period from 2000 to 2020. Temporary employment and unemployment (and, to some extent, self-employment) negatively influenced fertility more recently than in the past, particularly for second births (though differences were statistically significant only between the start and end of the observation period). Moreover, the role of employment status on women’s fertility began aligning with that of men, with temporary employment becoming increasingly detrimental to fertility in recent years for women (Alderotti et al., 2021, 2022); this is particularly evident in first- and second-order births. For men, changes in magnitude primarily pertained to unemployment and self-employment for first births, which seem less compatible with fatherhood. This finding appears to align with the changing composition of self-employed workers and the increasing precarisation of this group (Papa, 2021).

The documented changes in effect sizes appear unrelated to specific periods of crisis, which suggests a limited relevance of the economic business cycle. Our supplementary analyses further demonstrate that almost no change occurred in this time trend once we accounted for the varied composition of these groups in terms of occupational position and net salary, nor when we controlled for regional levels of youth unemployment (in quadratic form) as a potential confounder (Online Resources, Tables S2–S4).^9^ Thus, it appears that fertility decisions *are* related to individual-level employment instabilities, represented by non-permanent contracts and unemployment. Furthermore, these negative associations seem to become stronger over time, especially for women having their second child.

In our supplementary analyses, we found that the consequences of instability persisted even after accounting for economic and occupational situations, as captured by the salary deciles and social class of individual and their partner (Online Resources, Tables S2–S4). While there are limitations due to the measurement of economic situation and class, these findings contrast with van Wijk et al. (2021), who argued that the negative effects of temporary employment in the Netherlands were primarily due to lower incomes rather than employment instability; our results suggest that instability has a genuine effect independent of one’s salary or occupational position.

### Heterogeneous Effects of Employment Instability

In the following analysis, we examined the potential moderating effect of individuals’ education level on the relationship between employment instability and childbirth. Specifically, we investigated whether the effect of employment instability on fertility varies based on one’s level of education. Our initial hypothesis asserts that the negative association between employment instability and fertility may be more pronounced among highly educated individuals compared to those with a lower level of education, especially for women.

The results confirm that the effects of employment instability on having a first, second, and third child are not homogenous across individuals with different levels of education (Fig. 2). Aligning with our expectations, situations of unstable employment, and particularly unemployment, were (slightly) more inhibitory for fertility for those with higher levels of education. Temporary employment or unemployment, as opposed to permanent employment, was associated with much lower probabilities of having a first and second child, with this negative effect being more pronounced among highly educated individuals and less prominent among those with lower levels of education. These results hold for men and women, but contrary to our expectations, the results indicated a more pronounced moderation of the instability effects for men than for women: the difference in the probability of becoming a parent or having a second birth between low- and high-educated men with temporary contracts was much more pronounced than for women. The larger difference among men is driven by lower-educated men with temporary contracts experiencing a lesser impact on their likelihood of being parents compared to lower-educated women with temporary contracts. These results are also confirmed net of additional controls, including current salary and occupational position (Online Resources, Figure S1).

### Partner’s Employment Situation

Fertility is usually a decision between couples; thus, it not only requires a partner but is also influenced by the employment situation of both individuals involved. Results distinguishing the effects of employment status for those with and without a cohabiting partner are shown in ‘Appendix Fig. 6’. Interestingly, for women, we found that the employment-fertility nexus was much stronger in the presence of a male partner, especially for first births. This finding clearly demonstrates the relevance of women’s employment, in stark contrast to traditional notions of gender roles. For men, our measure cannot properly assess fertility for those who did not cohabit with their female partners, and the predicted probabilities for partnerless men to document childbirth were close to zero (not shown), independent of their employment situation.

Beyond the presence of a partner, his or her employment situation might be a relevant factor in the decision to have children. Figure 3 examines the extent to which the employment situation of one’s partner is important for fertility decisions, recognising that women’s employment within the couple has become more crucial for family formation. Figure 3 reports the estimates of the effect of ego’s and their partner’s employment situation on the likelihood of experiencing first, second, and higher births for both men and women. These estimates result from a joint model that considers the employment situation of one’s self and partner.

The partner’s employment situation is of relatively little importance for women, except that men’s inactivity hampers fertility. Instead, it is women’s own position that appears to make a difference. In line with this, for men, the partner’s situation plays a much more significant role than their own in reducing or encouraging fertility. As before, the effects are more pronounced for first and second births and almost absent for higher-order births.

**Fig. 7:**
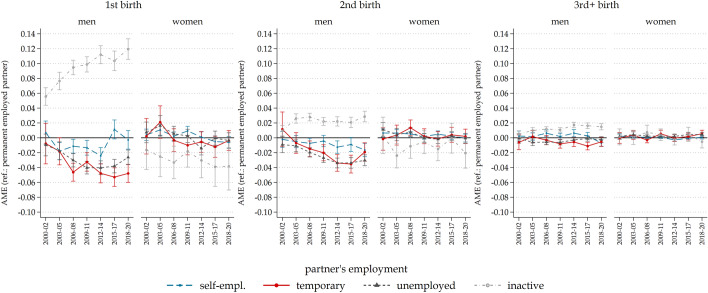
Childbirth and partner’s employment situation by sex, over time. *Source*: LFS, Italy 2000–2020. Average marginal effects (AME) from logistic regression models examining first, second and higher childbirth as a function of partner’s employment situation (1-year lagged) by sex and period, controlling for age, age-squared, level of education and ego’s employment situation (1-year lagged), with 95% confidence intervals

These findings suggest that women’s employment situation is decisive for fertility decisions, while men’s employment status is less relevant, independent of what their female partner does. Supplementary analyses show that when accounting for the economic situation (including zero incomes) of both partners as well as their occupational position (if any), the employment situation of women still appears to be decisive for fertility decisions, whereas, for men, the effect of temporary employment diminishes (Online Resources, Figure S2). Further analyses suggest that this is not a recent development, as the importance of the partner’s employment for women has been minimal since the beginning of our observation period in 2000. For men, the partner’s employment situation appears to have gained importance since the recession in 2007 (Fig. 7 ‘Appendix’). Thus, aligning with our expectations, the analyses show that the relevance of women’s employment situation for childbirth has increased over time.

## Robustness Checks

We conducted a series of robustness checks to ensure the validity of our results. First, we employed Poisson and complementary log–log regression as an alternative modelling technique to examine the relationship between employment instability and childbirth (Online Resources, Table S1, Model M0). Second, we examined the impact of different sample definitions on our results by conducting the analyses on alternative samples, including individuals older than 19 or older than 22, as well as restricting the analysis to those below the age of 40 (Online Resources, Table S1, Model M1-M3). Furthermore, considering the unique circumstances brought about by the COVID-19 pandemic in 2020, we conducted an analysis that excluded individuals observed in that year (Online Resources, Table S1, Model M4). Third, as extensively discussed in the text, we introduced controls for the net salary and social class of both the individual and their partner (if present) in some model specifications. This was done to separate employment instability from economic situations (Online Resources, Tables S2–S4, and Figures S1–S2), and the results seem to confirm the genuine relevance of instability. Additionally, we accounted for overall degrees of uncertainty by considering regional year-specific levels of youth unemployment (Online Resources, Tables S2–S4). None of these substantially altered the results, and the differences between permanent employment and unstable employment remained substantial throughout. Lastly, we explored various time specifications, including fully nonparametric, quadratic, and cubic specifications. The data clearly support the choice of a fully nonparametric model, where the inclusion of 3-year period dummies is sufficiently detailed. This solution was chosen as it preserves more information and aligns with the narrative that the 2003 and 2012 reforms were significant turning points in Italian history.

## Conclusion and Discussion

This paper offered four main contributions. First, it examined the effects of employment instability on fertility in Italy over a more extensive period than previous studies, testing for changes over time in these effects. Aligning with previous research, our study suggests that employment instability, such as temporary contracts and unemployment, correlates with lower fertility for both men and women. Furthermore, we show that, in Italy, the extent to which an individual’s employment instability hinders their fertility decisions has increased (H1 confirmed), specifically for women and, to a lesser, extent for men (H2 confirmed). We attribute this to the progression of labour market deregulation and the related dualisation, although we cannot fully rule out that these trends are also the result of broader social and cultural changes in reproductive behaviours. The general economic context (unemployment levels), however, does not seem to be responsible for time trends and the increasing importance of employment situations for fertility.

Second, we analysed the details regarding partners. Our findings suggest that women’s fertility choices substantially depend on their own employment instability (H3 confirmed). This challenges the traditional notion that the employment situation of the male partner is the main relevant factor for family formation or extension once a partnership is formed, and suggests that previous findings may have overstated the direct effect of the male partner’s instability. Concurrently, however, women’s inactivity remains an important predictor of childbearing, even more so for highly educated women. This is at odds with the idea that female employment nowadays is a precondition for parenthood. Notably, however, with women’s increasing labour market participation, those reporting inactivity appear to be increasingly more family-oriented. Thus, more detailed longitudinal data are indispensable to better understand this dynamic.

Third, the paper examined differences between social groups in terms of education level, highlighting that employment instability is more relevant for those with at least upper-level education (H4 confirmed). This could be attributed to different social expectations of parenthood or the fact that highly educated individuals may be more career-oriented.

Finally, we show that these effects are heterogeneous across parity progression (as expected in H5). Importantly, strong differences in childbirth between permanently employed people and those with unstable contracts were present not only for the likelihood of being parents, but also for the likelihood of having a second child, while they were almost absent for higher-order births.

Much of the analysis, including the examinations of change over time and (heterosexual) partner details, was made possible by the large number of cases in the LFS data, providing some exciting and partially novel results. Nevertheless, the data have some downsides that are worth repeating here. First, the measurement of fertility using the ‘own child method’ likely results in an underestimation of fertility for men. Second, the employment situation is only available for the year preceding the interview and 12 months prior to observing a newborn (i.e. a child below the age of one) in the household. This limitation restricts the study to exploring only short-term associations between employment status and fertility and poses some additional challenges, as some children may have been conceived before the employment status of the previous years was registered. However, as previous findings can be reproduced, there seems to be no major distortion caused by these limitations. We should also keep in mind that those with temporary employment in this analysis refer only to the more stable part of the non-permanent employed workforce, which implies that the negative effects of the contractual situation are likely underestimated, as it would reasonably be much stronger for those with fragmented careers and more precarious work positions. Finally, the lack of longitudinal information prevented us from making any claim regarding whether employment instability is associated with delayed or foregone fertility.

Overall, this study sheds new light on a socially relevant question, highlighting that the employment situation of women deserves more extensive attention to help aid one’s reproductive decisions. We find clear indications that women’s employment situation can no longer be considered as ‘secondary’. Future research should extend these findings and focus on the partnership-formation process and employment dynamics within the couple. Beyond addressing the importance of pooling socio-economic resources, a focus on both partners’ occupations is central to highlighting potential mechanisms associated with the changing roles of women in society.

### Electronic supplementary material

Below is the link to the electronic supplementary material.

Supplementary file1 (DOCX 455 kb)

## Figures and Tables

**Fig. 4:**
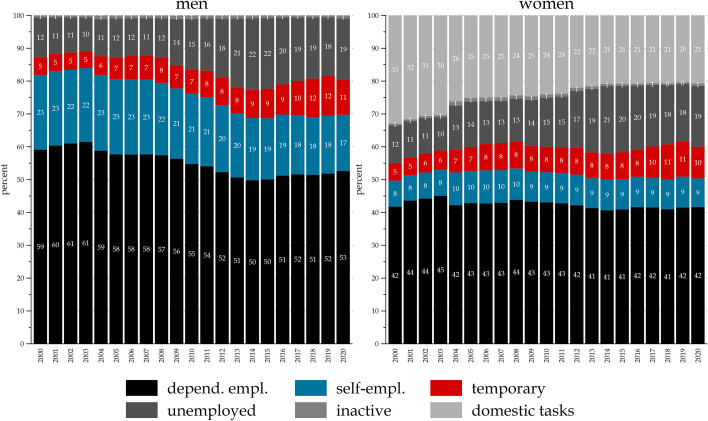
Main employment situation of men and women (age 15–49), Italy 2000–2020. *Source*: LFS, Italy 2000–2020, weighted statistics. See text for sample description

**Fig. 5:**
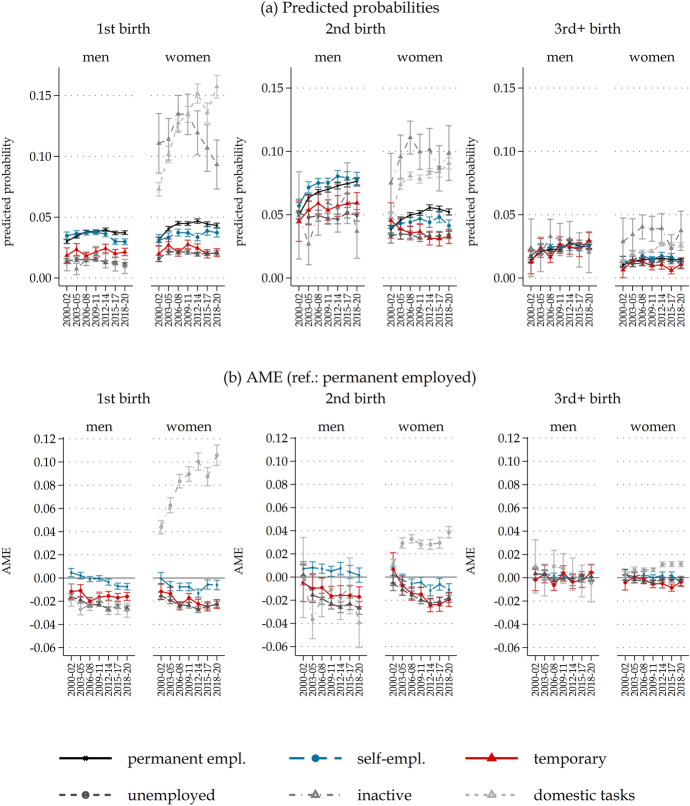
Childbirth and employment situation over time, including inactive. *Source*: LFS, Italy 2000–2020. *Note*: Predicted probabilities (**A**) and average marginal effects (**B**) from logistic regression models examining first, second, and higher childbirth as a function of employment situation (1-year lagged) by sex and period. The results are based on nonparametric time trends and adjusted for age, age-squared, and level of education, and include 95% confidence intervals

**Fig. 6:**
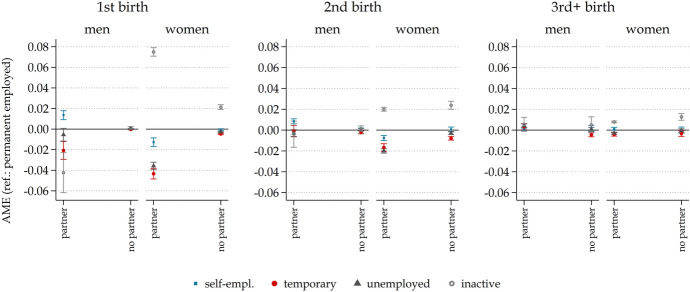
Childbirth and employment situation, by partnership status and sex. *Source*: LFS, Italy 2000–2020. *Note*: Average marginal effects (AME) from logistic regression models examining first, second, and higher childbirth as a function of employment situation (1-year lagged) by sex and presence of a cohabiting partner, controlling for age, age–squared, period, and level of education, with 95% confidence intervals

## Appendix

See Figs. 4, 5, 6, 7 and Table 1.

**Table 1:** Descriptive sample statistics, by sex

	Men	Women	Total
1st birth	3.42	4.58	3.93
2nd birth	6.20	5.50	5.82
3rd + birth	2.00	1.80	1.89
Employment situation (1-year lagged)			
Permanent empl	56.93	44.00	50.52
Self-empl	21.27	10.06	15.71
Temporary	3.96	3.94	3.95
Unemployed	16.89	16.35	16.62
Inactive	0.94	0.94	0.94
Domestic tasks	0.00	24.72	12.26
Educational attainment			
Low: Lower secondary or below	42.29	36.93	39.64
Medium: Upper secondary	45.08	45.42	45.25
High: Third level	12.63	17.65	15.12
Age			
15–19	1.41	0.98	1.20
20–24	8.19	6.74	7.47
25–29	13.52	13.30	13.41
30–34	17.65	17.85	17.75
35–39	19.65	20.03	19.84
40–44	20.20	20.83	20.51
45–49	19.38	20.27	19.82
Social Class (ESeG)			
Upper	12.16	11.87	12.01
Mid	63.63	40.13	52.00
Low	17.10	22.39	19.72
Not employed	7.11	25.61	16.27
Income (monthly pay from main job)			
Below the 1st decile	5.39	16.75	10.45
1st–2nd decile	7.15	15.59	10.91
2nd–3rd decile	9.31	13.30	11.09
3rd–4th decile	10.39	10.88	10.61
4th–5th decile	11.55	9.78	10.76
5th–6th decile	10.98	8.73	9.98
6th–7th decile	11.26	8.07	9.84
7th–8th decile	11.42	7.11	9.50
8th–9th decile	11.46	5.54	8.82
More or equal to the 9th decile	11.08	4.26	8.04
Without a partner	44.71	33.22	39.02
Employment situation of the partner (1-year lagged)			
Permanent. empl	42.80	62.67	53.53
Self-empl	9.87	24.73	17.89
Temporary	3.04	2.60	2.80
Unemployed	11.12	9.03	10.00
Inactive	33.17	0.96	15.79
Partner’s income (monthly pay from main job)			
Below the 1st decile	15.58	3.10	7.87
1st–2nd decile	14.92	4.42	8.43
2nd–3rd decile	12.65	6.52	8.86
3rd–4th decile	10.60	8.24	9.14
4th–5th decile	9.87	10.58	10.31
5th–6th decile	9.33	11.12	10.44
6th–7th decile	8.75	12.49	11.06
7th–8th decile	7.69	13.56	11.32
8th–9th decile	6.04	14.55	11.30
More or equal to the 9th decile	4.57	15.42	11.27
Observations	1,758,467	1,792,328	3,550,795

*Source*: LFS, Italy 2000–2020, weighted statistics

## Footnotes

1 There was an attempt to partially reregulate their use in 2012 (Riforma Fornero-Monti, Law No. 92/2012). However, it was soon abandoned by the subsequent governments.

2 Therefore, we could not investigate in detail the mediating role of partnership formation for childbirth, although we could still argue for examining the situation regarding socially fathering a child.

3 Before 2004, only children up to age 15 are reported and, afterwards, only for those up to 25.

4 Changes in the employment situation from one year to another illustrate the following picture: over the 20 years, about 92% of the individuals did not change their work status (employed – not employed), while between 2% (in 2000) and 5% (in 2020) stopped working from one year to another. The latter increase was gradual, and between 5 and 6% started working from one year to another.

5 Employment is defined as follows: ‘carries out a job or profession, including unpaid work for a family business or holding, including an apprenticeship or paid traineeship, etc.’. The distinction between self-employment and dependent employment comes from the professional status. Self-employment also includes family workers.

6 In detail, temporary employment the previous year was differentiated from current employment contracts that were conditional to start prior to 12 months. This definition underestimates the share of temporary contracts and limits the measure to those who remained in a temporary employment situation for at least a year. The interpretation will have to bear in mind that it refers to the stable part of fixed-term employment. Moreover, it implies that we underestimated the share and effects of temporary instability, which is acceptable in a conservative perspective that wishes to test for differences.

7 These include: Managers and professionals for the upper class; technicians and professional employees, small entrepreneurs, clerks, and skilled service and industrial workers for the middle class; and lower status or unskilled workers for the lower class. The categorical measure contains a category for all those outside the labour market.

8 Adding a control for the change in employment position did not alter the results.

9 Additional analysis (not reported) suggests that the negative effects of temporary employment on fertility diminish once uncertainties become more generalised, such as with higher levels of unemployment. This finding implies that the relative differences between employment contracts diminish as risks of employment-loss increase.
