# Supplementary material for: Employment Instability and Childbirth over the Last 20 Years in Italy
Source: Eur J Popul. 2023 Oct 12;39(1):31. doi: 10.1007/s10680-023-09680-5 (PMC10570255; doi:10.1007/s10680-023-09680-5)
Supplement: Supplementary file 1 — Supplementary file1 (DOCX 455 kb) [file 10680_2023_9680_MOESM1_ESM.docx]

**Supplementary Information**

Employment Instability and Childbirth Over the Last 20 Years in Italy

Journal: European Journal of Population

# **Table S1** AMEs from different models’ specifications examining first, second, and higher childbirths as a function of employment situation (1-year lagged). 95% confidence intervals in brackets.

**Men**

|  | M0 (Poisson) | M1 (age 15–39) | M2 (age 20–49) | M3 (age 20–44) | M4 (pre-2020) | M0 (Poisson) | M1 (age 15–39) | M2 (age 20–49) | M3 (age 20–44) | M4 (pre-2020) | M0 (Poisson) | M1 (age 15–39) | M2 (age 20–49) | M3 (age 20–44) | M4 (pre-2020) |
| --- | --- | --- | --- | --- | --- | --- | --- | --- | --- | --- | --- | --- | --- | --- | --- |
|  | 1st Birth | 1st Birth | 1st Birth | 1st Birth | 1st Birth | 2nd Birth | 2nd Birth | 2nd Birth | 2nd Birth | 2nd Birth | 3rd Birth | 3rd Birth | 3rd Birth | 3rd Birth | 3rd Birth |
| Self-empl × 2000 | 0.005 | 0.007 | 0.004 | 0.006 | 0.005 | 0.007 | 0.012 | 0.007 | 0.009 | 0.007 | 0.003 | 0.003 | 0.003 | 0.004 | 0.003 |
|  | [0.001,  0.008] | [0.002,  0.012] | [0.001,  0.008] | [0.002,  0.010] | [0.001,  0.008] | [0.001,  0.013] | [0.002,  0.022] | [0.001,  0.014] | [0.002,  0.017] | [0.001,  0.013] | [–0.000,  0.007] | [–0.003,  0.009] | [–0.000,  0.007] | [–0.001,  0.008] | [–0.000,  0.007] |
| Self-empl × 2003 | 0.002 | 0.001 | 0.002 | 0.002 | 0.002 | 0.008 | 0.011 | 0.008 | 0.010 | 0.008 | 0.003 | 0.005 | 0.003 | 0.004 | 0.003 |
|  | [–0.001,  0.005] | [–0.002,  0.005] | [–0.001,  0.005] | [–0.002,  0.005] | [–0.001,  0.005] | [0.003,  0.013] | [0.003,  0.019] | [0.003,  0.013] | [0.003,  0.016] | [0.003,  0.013] | [0.000,  0.006] | [–0.001,  0.011] | [–0.000,  0.006] | [0.000,  0.008] | [0.000,  0.006] |
| Self-empl × 2006 | –0.001 | –0.003 | –0.001 | –0.002 | –0.001 | 0.007 | 0.009 | 0.007 | 0.010 | 0.007 | 0.002 | 0.001 | 0.002 | 0.003 | 0.002 |
|  | [–0.003,  0.001] | [–0.006,  –0.000] | [–0.003,  0.001] | [–0.004,  0.001] | [–0.003,  0.001] | [0.003,  0.011] | [0.003,  0.016] | [0.003,  0.011] | [0.004,  0.015] | [0.003,  0.011] | [–0.001,  0.004] | [–0.003,  0.006] | [–0.001,  0.004] | [–0.000,  0.006] | [–0.001,  0.004] |
| Self-empl × 2009 | –0.000 | –0.002 | –0.000 | –0.001 | –0.000 | 0.005 | 0.003 | 0.005 | 0.006 | 0.005 | 0.002 | 0.001 | 0.002 | 0.002 | 0.002 |
|  | [–0.003,  0.002] | [–0.005,  0.001] | [–0.003,  0.002] | [–0.003,  0.002] | [–0.003,  0.002] | [0.001,  0.009] | [–0.004,  0.010] | [0.000,  0.009] | [0.000,  0.011] | [0.001,  0.009] | [–0.000,  0.005] | [–0.004,  0.005] | [–0.000,  0.005] | [–0.001,  0.005] | [–0.000,  0.005] |
| Self-empl × 2012 | –0.004 | –0.007 | –0.004 | –0.005 | –0.004 | 0.008 | 0.005 | 0.008 | 0.011 | 0.008 | –0.004 | –0.006 | –0.004 | –0.005 | –0.004 |
|  | [–0.006,  –0.001] | [–0.011,  –0.004] | [–0.006,  –0.001] | [–0.008,  –0.002] | [–0.006,  –0.001] | [0.003,  0.013] | [–0.002,  0.013] | [0.003,  0.013] | [0.004,  0.018] | [0.003,  0.013] | [–0.007,  –0.001] | [–0.012,  –0.001] | [–0.007,  –0.001] | [–0.009,  –0.001] | [–0.007,  –0.001] |
| Self-empl × 2015 | –0.007 | –0.010 | –0.007 | –0.008 | –0.007 | 0.004 | 0.004 | 0.004 | 0.006 | 0.004 | –0.001 | –0.002 | –0.001 | –0.001 | –0.001 |
|  | [–0.010,  –0.004] | [–0.013,  –0.006] | [–0.010,  –0.004] | [–0.011,  –0.005] | [–0.010,  –0.004] | [–0.001,  0.010] | [–0.005,  0.013] | [–0.001,  0.010] | [–0.002,  0.013] | [–0.001,  0.010] | [–0.004,  0.002] | [–0.009,  0.004] | [–0.004,  0.002] | [–0.005,  0.003] | [–0.004,  0.002] |
| Self-empl × 2018 | –0.007 | –0.011 | –0.008 | –0.010 | –0.007 | 0.002 | 0.001 | 0.002 | –0.003 | 0.000 | 0.000 | 0.000 | 0.000 | 0.002 | 0.002 |
|  | [–0.010,  –0.005] | [–0.015,  –0.007] | [–0.010,  –0.005] | [–0.013,  –0.007] | [–0.011,  –0.004] | [–0.005,  0.008] | [–0.008,  0.011] | [–0.005,  0.008] | [–0.010,  0.005] | [–0.007,  0.008] | [–0.003,  0.004] | [–0.007,  0.007] | [–0.003,  0.004] | [–0.002,  0.007] | [–0.003,  0.006] |
| Temporary × 2000 | –0.012 | –0.016 | –0.011 | –0.013 | –0.012 | –0.005 | –0.001 | –0.006 | –0.005 | –0.005 | –0.002 | –0.002 | –0.001 | –0.001 | –0.002 |
|  | [–0.018,  –0.005] | [–0.025,  –0.008] | [–0.018,  –0.005] | [–0.021,  –0.005] | [–0.018,  –0.005] | [–0.021,  0.011] | [–0.027,  0.026] | [–0.022,  0.010] | [–0.026,  0.016] | [–0.021,  0.011] | [–0.011,  0.008] | [–0.018,  0.014] | [–0.011,  0.008] | [–0.013,  0.011] | [–0.011,  0.008] |
| Temporary × 2003 | –0.011 | –0.016 | –0.011 | –0.015 | –0.011 | –0.010 | –0.011 | –0.010 | –0.012 | –0.010 | 0.003 | 0.006 | 0.004 | 0.006 | 0.003 |
|  | [–0.016,  –0.006] | [–0.023,  –0.010] | [–0.016,  –0.006] | [–0.021,  –0.009] | [–0.016,  –0.006] | [–0.022,  0.002] | [–0.028,  0.007] | [–0.022,  0.003] | [–0.027,  0.003] | [–0.022,  0.002] | [–0.004,  0.011] | [–0.007,  0.020] | [–0.004,  0.012] | [–0.004,  0.015] | [–0.004,  0.011] |
| Temporary × 2006 | –0.020 | –0.026 | –0.020 | –0.024 | –0.020 | –0.009 | –0.012 | –0.008 | –0.012 | –0.009 | –0.006 | –0.012 | –0.006 | –0.008 | –0.006 |
|  | [–0.023,  –0.017] | [–0.030,  –0.022] | [–0.023,  –0.017] | [–0.028,  –0.020] | [–0.023,  –0.017] | [–0.018,  –0.000] | [–0.024,  0.001] | [–0.017,  0.001] | [–0.022,  –0.001] | [–0.018,  –0.001] | [–0.011,  –0.002] | [–0.020,  –0.003] | [–0.011,  –0.002] | [–0.014,  –0.002] | [–0.011,  –0.002] |
| Temporary × 2009 | –0.017 | –0.021 | –0.016 | –0.020 | –0.017 | –0.016 | –0.027 | –0.016 | –0.021 | –0.016 | 0.004 | 0.004 | 0.005 | 0.003 | 0.004 |
|  | [–0.020,  –0.013] | [–0.026,  –0.017] | [–0.020,  –0.013] | [–0.024,  –0.015] | [–0.020,  –0.013] | [–0.025,  –0.008] | [–0.039,  –0.016] | [–0.024,  –0.007] | [–0.032,  –0.011] | [–0.025,  –0.008] | [–0.002,  0.010] | [–0.007,  0.015] | [–0.002,  0.011] | [–0.005,  0.010] | [–0.002,  0.010] |
| Temporary × 2012 | –0.015 | –0.017 | –0.015 | –0.017 | –0.015 | –0.016 | –0.014 | –0.015 | –0.019 | –0.016 | –0.003 | –0.006 | –0.003 | –0.005 | –0.003 |
|  | [–0.019,  –0.012] | [–0.023,  –0.012] | [–0.019,  –0.011] | [–0.022,  –0.013] | [–0.019,  –0.012] | [–0.025,  –0.007] | [–0.027,  –0.001] | [–0.024,  –0.006] | [–0.029,  –0.008] | [–0.025,  –0.008] | [–0.009,  0.003] | [–0.017,  0.005] | [–0.009,  0.003] | [–0.012,  0.003] | [–0.009,  0.003] |
| Temporary × 2015 | –0.017 | –0.019 | –0.017 | –0.018 | –0.017 | –0.016 | –0.016 | –0.015 | –0.018 | –0.016 | –0.003 | –0.009 | –0.002 | –0.003 | –0.003 |
|  | [–0.020,  –0.013] | [–0.024,  –0.014] | [–0.020,  –0.013] | [–0.023,  –0.014] | [–0.021,  –0.013] | [–0.025,  –0.007] | [–0.028,  –0.003] | [–0.024,  –0.006] | [–0.028,  –0.007] | [–0.025,  –0.007] | [–0.008,  0.003] | [–0.019,  0.000] | [–0.008,  0.004] | [–0.010,  0.005] | [–0.008,  0.003] |
| Temporary × 2018 | –0.016 | –0.017 | –0.016 | –0.018 | –0.016 | –0.017 | –0.022 | –0.016 | –0.020 | –0.010 | 0.005 | –0.007 | 0.005 | 0.005 | 0.008 |
|  | [–0.019,  –0.012] | [–0.021,  –0.012] | [–0.019,  –0.012] | [–0.022,  –0.014] | [–0.020,  –0.012] | [–0.026,  –0.008] | [–0.034,  –0.010] | [–0.025,  –0.007] | [–0.031,  –0.010] | [–0.021,  0.001] | [–0.002,  0.011] | [–0.017,  0.003] | [–0.002,  0.012] | [–0.003,  0.013] | [–0.000,  0.016] |
| Unemployed × 2000 | –0.016 | –0.021 | –0.017 | –0.019 | –0.016 | 0.002 | 0.002 | –0.001 | 0.002 | 0.002 | 0.004 | 0.008 | 0.004 | 0.004 | 0.004 |
|  | [–0.019,  –0.013] | [–0.024,  –0.017] | [–0.020,  –0.014] | [–0.022,  –0.015] | [–0.019,  –0.013] | [–0.007,  0.010] | [–0.011,  0.015] | [–0.009,  0.008] | [–0.009,  0.013] | [–0.007,  0.010] | [–0.002,  0.009] | [–0.001,  0.017] | [–0.002,  0.009] | [–0.002,  0.011] | [–0.002,  0.009] |
| Unemployed × 2003 | –0.019 | –0.024 | –0.019 | –0.023 | –0.019 | –0.016 | –0.024 | –0.018 | –0.022 | –0.016 | 0.003 | 0.006 | 0.002 | 0.004 | 0.003 |
|  | [–0.021,  –0.017] | [–0.027,  –0.021] | [–0.022,  –0.017] | [–0.026,  –0.020] | [–0.021,  –0.017] | [–0.022,  –0.009] | [–0.033,  –0.015] | [–0.024,  –0.011] | [–0.030,  –0.014] | [–0.022,  –0.009] | [–0.002,  0.007] | [–0.002,  0.014] | [–0.002,  0.007] | [–0.001,  0.009] | [–0.002,  0.007] |
| Unemployed × 2006 | –0.023 | –0.030 | –0.024 | –0.028 | –0.023 | –0.019 | –0.028 | –0.019 | –0.024 | –0.019 | –0.002 | –0.004 | –0.002 | –0.004 | –0.003 |
|  | [–0.025,  –0.022] | [–0.032,  –0.027] | [–0.026,  –0.022] | [–0.030,  –0.026] | [–0.025,  –0.022] | [–0.024,  –0.014] | [–0.035,  –0.021] | [–0.024,  –0.015] | [–0.029,  –0.018] | [–0.024,  –0.014] | [–0.005,  0.000] | [–0.009,  0.001] | [–0.005,  0.001] | [–0.008,  –0.001] | [–0.005,  0.000] |
| Unemployed × 2009 | –0.023 | –0.029 | –0.023 | –0.027 | –0.023 | –0.023 | –0.034 | –0.024 | –0.028 | –0.023 | –0.000 | 0.000 | –0.000 | –0.000 | –0.000 |
|  | [–0.025,  –0.021] | [–0.031,  –0.026] | [–0.025,  –0.021] | [–0.029,  –0.025] | [–0.025,  –0.021] | [–0.028,  –0.018] | [–0.041,  –0.027] | [–0.028,  –0.019] | [–0.034,  –0.023] | [–0.028,  –0.018] | [–0.003,  0.003] | [–0.005,  0.006] | [–0.003,  0.003] | [–0.004,  0.004] | [–0.003,  0.003] |
| Unemployed × 2012 | –0.027 | –0.033 | –0.027 | –0.031 | –0.027 | –0.026 | –0.038 | –0.026 | –0.033 | –0.026 | –0.000 | –0.003 | 0.000 | –0.000 | –0.000 |
|  | [–0.029,  –0.025] | [–0.035,  –0.030] | [–0.029,  –0.025] | [–0.033,  –0.029] | [–0.029,  –0.025] | [–0.031,  –0.022] | [–0.045,  –0.032] | [–0.031,  –0.021] | [–0.038,  –0.027] | [–0.031,  –0.022] | [–0.003,  0.003] | [–0.010,  0.003] | [–0.003,  0.004] | [–0.005,  0.004] | [–0.004,  0.003] |
| Unemployed × 2015 | –0.024 | –0.030 | –0.025 | –0.028 | –0.024 | –0.023 | –0.025 | –0.023 | –0.028 | –0.023 | 0.002 | 0.001 | 0.002 | 0.001 | 0.002 |
|  | [–0.026,  –0.023] | [–0.032,  –0.027] | [–0.027,  –0.023] | [–0.030,  –0.026] | [–0.026,  –0.023] | [–0.028,  –0.018] | [–0.032,  –0.018] | [–0.028,  –0.018] | [–0.034,  –0.022] | [–0.028,  –0.018] | [–0.002,  0.005] | [–0.005,  0.007] | [–0.002,  0.005] | [–0.003,  0.006] | [–0.002,  0.005] |
| Unemployed × 2018 | –0.026 | –0.032 | –0.026 | –0.031 | –0.027 | –0.027 | –0.040 | –0.027 | –0.033 | –0.030 | 0.001 | 0.001 | 0.001 | 0.001 | 0.001 |
|  | [–0.028,  –0.024] | [–0.034,  –0.029] | [–0.028,  –0.024] | [–0.033,  –0.028] | [–0.029,  –0.025] | [–0.032,  –0.021] | [–0.048,  –0.033] | [–0.032,  –0.021] | [–0.039,  –0.026] | [–0.036,  –0.023] | [–0.003,  0.005] | [–0.006,  0.008] | [–0.003,  0.005] | [–0.003,  0.006] | [–0.004,  0.005] |
| Inactive × 2000 | –0.015 | –0.022 | –0.015 | –0.019 | –0.015 | –0.000 | 0.005 | 0.001 | 0.002 | –0.000 | 0.010 | 0.029 | 0.011 | 0.013 | 0.010 |
|  | [–0.024,  –0.005] | [–0.034,  –0.009] | [–0.025,  –0.005] | [–0.030,  –0.008] | [–0.024,  –0.005] | [–0.035,  0.035] | [–0.053,  0.062] | [–0.035,  0.037] | [–0.043,  0.046] | [–0.035,  0.034] | [–0.013,  0.033] | [–0.017,  0.075] | [–0.013,  0.036] | [–0.016,  0.043] | [–0.013,  0.033] |
| Inactive × 2003 | –0.027 | –0.034 | –0.027 | –0.031 | –0.027 | –0.037 | –0.054 | –0.037 | –0.051 | –0.037 | –0.002 | –0.000 | –0.001 | 0.000 | –0.002 |
|  | [–0.032,  –0.022] | [–0.040,  –0.028] | [–0.032,  –0.022] | [–0.037,  –0.026] | [–0.032,  –0.022] | [–0.053,  –0.020] | [–0.078,  –0.030] | [–0.054,  –0.020] | [–0.070,  –0.032] | [–0.053,  –0.020] | [–0.016,  0.012] | [–0.025,  0.025] | [–0.015,  0.013] | [–0.018,  0.018] | [–0.016,  0.012] |
| Inactive × 2006 | –0.023 | –0.027 | –0.024 | –0.028 | –0.023 | –0.018 | –0.028 | –0.017 | –0.025 | –0.018 | 0.010 | 0.010 | 0.009 | 0.008 | 0.010 |
|  | [–0.028,  –0.018] | [–0.035,  –0.020] | [–0.029,  –0.019] | [–0.034,  –0.021] | [–0.028,  –0.018] | [–0.034,  –0.001] | [–0.052,  –0.004] | [–0.034,  –0.000] | [–0.045,  –0.004] | [–0.034,  –0.002] | [–0.004,  0.024] | [–0.014,  0.034] | [–0.004,  0.023] | [–0.009,  0.024] | [–0.004,  0.024] |
| Inactive × 2009 | –0.018 | –0.024 | –0.019 | –0.022 | –0.018 | –0.011 | –0.007 | –0.013 | –0.013 | –0.011 | 0.008 | 0.011 | 0.007 | 0.012 | 0.008 |
|  | [–0.024,  –0.012] | [–0.033,  –0.016] | [–0.025,  –0.013] | [–0.029,  –0.014] | [–0.024,  –0.012] | [–0.028,  0.007] | [–0.033,  0.020] | [–0.030,  0.004] | [–0.034,  0.009] | [–0.028,  0.006] | [–0.005,  0.021] | [–0.012,  0.035] | [–0.006,  0.020] | [–0.005,  0.029] | [–0.005,  0.021] |
| Inactive × 2012 | –0.026 | –0.033 | –0.027 | –0.030 | –0.026 | –0.018 | –0.042 | –0.017 | –0.024 | –0.018 | 0.003 | 0.008 | 0.004 | 0.004 | 0.003 |
|  | [–0.031,  –0.020] | [–0.041,  –0.026] | [–0.033,  –0.021] | [–0.037,  –0.023] | [–0.031,  –0.020] | [–0.037,  0.002] | [–0.067,  –0.017] | [–0.036,  0.003] | [–0.048,  –0.001] | [–0.037,  0.001] | [–0.011,  0.017] | [–0.020,  0.036] | [–0.011,  0.018] | [–0.014,  0.021] | [–0.011,  0.017] |
| Inactive × 2015 | –0.025 | –0.031 | –0.026 | –0.029 | –0.025 | –0.008 | 0.001 | –0.007 | –0.007 | –0.008 | –0.003 | 0.000 | –0.002 | –0.003 | –0.003 |
|  | [–0.031,  –0.020] | [–0.039,  –0.023] | [–0.032,  –0.020] | [–0.036,  –0.022] | [–0.031,  –0.019] | [–0.032,  0.017] | [–0.037,  0.039] | [–0.032,  0.018] | [–0.037,  0.024] | [–0.032,  0.016] | [–0.016,  0.011] | [–0.028,  0.029] | [–0.016,  0.011] | [–0.021,  0.015] | [–0.016,  0.011] |
| Inactive × 2018 | –0.028 | –0.032 | –0.028 | –0.033 | –0.027 | –0.039 | –0.047 | –0.039 | –0.049 | –0.036 | –0.005 | –0.006 | –0.004 | –0.007 | –0.005 |
|  | [–0.034,  –0.022] | [–0.040,  –0.023] | [–0.034,  –0.022] | [–0.040,  –0.026] | [–0.035,  –0.019] | [–0.061,  –0.018] | [–0.080,  –0.014] | [–0.061,  –0.017] | [–0.076,  –0.022] | [–0.063,  –0.009] | [–0.021,  0.012] | [–0.035,  0.023] | [–0.021,  0.013] | [–0.027,  0.013] | [–0.023,  0.014] |
| Observations | 1569714 | 1043704 | 1549171 | 1280440 | 1504401 | 1005919 | 558347 | 990800 | 772975 | 966535 | 1088144 | 474249 | 1081349 | 818502 | 1042702 |

(continues on the next page)

(continues from the previous page)

**Women**

|  | M0 (poisson) | M1 (age 15–39) | M2 (age 20–49) | M3 (age 20–44) | M4 (pre-2020) | M0 (poisson) | M1 (age 15–39) | M2 (age 20–49) | M3 (age 20–44) | M4 (pre-2020) | M0 (poisson) | M1 (age 15–39) | M2 (age 20–49) | M3 (age 20–44) | M4 (pre-2020) |
| --- | --- | --- | --- | --- | --- | --- | --- | --- | --- | --- | --- | --- | --- | --- | --- |
|  | 1st Birth | 1st Birth | 1st Birth | 1st Birth | 1st Birth | 2nd Birth | 2nd Birth | 2nd Birth | 2nd Birth | 2nd Birth | 3rd Birth | 3rd Birth | 3rd Birth | 3rd Birth | 3rd Birth |
| Self-empl × 2000 | –0.000 | 0.001 | –0.000 | –0.001 | –0.000 | 0.002 | 0.003 | 0.002 | 0.002 | 0.002 | 0.002 | 0.002 | 0.002 | 0.002 | 0.002 |
|  | [–0.005,  0.005] | [–0.007,  0.009] | [–0.006,  0.005] | [–0.007,  0.006] | [–0.005,  0.005] | [–0.005,  0.009] | [–0.008,  0.015] | [–0.005,  0.009] | [–0.007,  0.011] | [–0.005,  0.009] | [–0.002,  0.007] | [–0.006,  0.010] | [–0.002,  0.007] | [–0.003,  0.008] | [–0.002,  0.007] |
| Self-empl × 2003 | –0.007 | –0.009 | –0.007 | –0.009 | –0.007 | –0.003 | –0.005 | –0.003 | –0.003 | –0.003 | 0.001 | 0.001 | 0.001 | 0.001 | 0.001 |
|  | [–0.011,  –0.003] | [–0.015,  –0.003] | [–0.011,  –0.003] | [–0.014,  –0.004] | [–0.011,  –0.003] | [–0.008,  0.003] | [–0.014,  0.004] | [–0.008,  0.003] | [–0.010,  0.004] | [–0.008,  0.003] | [–0.002,  0.004] | [–0.005,  0.007] | [–0.002,  0.004] | [–0.003,  0.005] | [–0.002,  0.004] |
| Self-empl × 2006 | –0.008 | –0.010 | –0.008 | –0.009 | –0.008 | –0.006 | –0.010 | –0.006 | –0.007 | –0.006 | 0.003 | 0.004 | 0.003 | 0.004 | 0.003 |
|  | [–0.011,  –0.004] | [–0.015,  –0.006] | [–0.011,  –0.005] | [–0.013,  –0.005] | [–0.011,  –0.004] | [–0.010,  –0.002] | [–0.017,  –0.003] | [–0.010,  –0.002] | [–0.012,  –0.002] | [–0.010,  –0.002] | [0.001,  0.006] | [–0.001,  0.010] | [0.001,  0.006] | [0.000,  0.007] | [0.001,  0.006] |
| Self-empl × 2009 | –0.008 | –0.011 | –0.008 | –0.009 | –0.008 | –0.004 | –0.008 | –0.005 | –0.006 | –0.004 | –0.000 | 0.001 | 0.000 | 0.000 | 0.000 |
|  | [–0.011,  –0.004] | [–0.016,  –0.006] | [–0.011,  –0.004] | [–0.014,  –0.005] | [–0.011,  –0.004] | [–0.009,  –0.000] | [–0.015,  –0.000] | [–0.009,  –0.000] | [–0.012,  –0.000] | [–0.009,  –0.000] | [–0.003,  0.003] | [–0.005,  0.006] | [–0.002,  0.003] | [–0.003,  0.003] | [–0.003,  0.003] |
| Self-empl × 2012 | –0.013 | –0.017 | –0.013 | –0.015 | –0.013 | –0.011 | –0.015 | –0.012 | –0.015 | –0.012 | 0.002 | 0.003 | 0.002 | 0.003 | 0.002 |
|  | [–0.017,  –0.010] | [–0.022,  –0.012] | [–0.017,  –0.010] | [–0.020,  –0.011] | [–0.017,  –0.010] | [–0.016,  –0.007] | [–0.023,  –0.007] | [–0.016,  –0.007] | [–0.021,  –0.008] | [–0.016,  –0.007] | [–0.001,  0.005] | [–0.003,  0.009] | [–0.001,  0.005] | [–0.001,  0.006] | [–0.001,  0.005] |
| Self-empl × 2015 | –0.006 | –0.008 | –0.006 | –0.007 | –0.006 | –0.006 | –0.011 | –0.006 | –0.008 | –0.006 | 0.002 | 0.002 | 0.002 | 0.002 | 0.002 |
|  | [–0.010,  –0.002] | [–0.014,  –0.002] | [–0.010,  –0.002] | [–0.012,  –0.002] | [–0.010,  –0.002] | [–0.011,  –0.001] | [–0.020,  –0.002] | [–0.011,  –0.001] | [–0.014,  –0.001] | [–0.011,  –0.001] | [–0.001,  0.005] | [–0.005,  0.008] | [–0.001,  0.005] | [–0.002,  0.006] | [–0.001,  0.005] |
| Self-empl × 2018 | –0.006 | –0.009 | –0.006 | –0.007 | –0.006 | –0.011 | –0.018 | –0.011 | –0.014 | –0.017 | –0.004 | –0.009 | –0.004 | –0.005 | –0.003 |
|  | [–0.010,  –0.002] | [–0.015,  –0.003] | [–0.010,  –0.002] | [–0.012,  –0.002] | [–0.011,  –0.001] | [–0.016,  –0.006] | [–0.027,  –0.009] | [–0.016,  –0.006] | [–0.020,  –0.007] | [–0.023,  –0.011] | [–0.007,  –0.001] | [–0.015,  –0.003] | [–0.007,  –0.001] | [–0.009,  –0.001] | [–0.006,  0.001] |
| Temporary × 2000 | –0.012 | –0.019 | –0.011 | –0.014 | –0.012 | 0.007 | 0.011 | 0.006 | 0.009 | 0.007 | –0.004 | –0.006 | –0.004 | –0.005 | –0.004 |
|  | [–0.018,  –0.005] | [–0.029,  –0.010] | [–0.018,  –0.005] | [–0.023,  –0.006] | [–0.018,  –0.005] | [–0.007,  0.021] | [–0.012,  0.035] | [–0.008,  0.020] | [–0.009,  0.027] | [–0.007,  0.021] | [–0.011,  0.003] | [–0.018,  0.006] | [–0.011,  0.003] | [–0.013,  0.003] | [–0.011,  0.003] |
| Temporary × 2003 | –0.013 | –0.020 | –0.013 | –0.017 | –0.014 | –0.007 | –0.011 | –0.007 | –0.010 | –0.007 | 0.000 | 0.001 | 0.001 | 0.001 | 0.000 |
|  | [–0.019,  –0.008] | [–0.028,  –0.013] | [–0.019,  –0.008] | [–0.023,  –0.010] | [–0.019,  –0.008] | [–0.016,  0.001] | [–0.025,  0.003] | [–0.016,  0.001] | [–0.020,  0.001] | [–0.016,  0.001] | [–0.004,  0.005] | [–0.008,  0.010] | [–0.004,  0.005] | [–0.005,  0.007] | [–0.004,  0.005] |
| Temporary × 2006 | –0.024 | –0.034 | –0.024 | –0.029 | –0.024 | –0.014 | –0.025 | –0.014 | –0.019 | –0.014 | –0.002 | –0.005 | –0.002 | –0.002 | –0.002 |
|  | [–0.027,  –0.020] | [–0.039,  –0.029] | [–0.028,  –0.020] | [–0.034,  –0.025] | [–0.027,  –0.020] | [–0.020,  –0.009] | [–0.035,  –0.016] | [–0.020,  –0.008] | [–0.027,  –0.012] | [–0.020,  –0.009] | [–0.005,  0.002] | [–0.012,  0.001] | [–0.005,  0.002] | [–0.007,  0.002] | [–0.005,  0.002] |
| Temporary × 2009 | –0.017 | –0.025 | –0.017 | –0.021 | –0.017 | –0.015 | –0.023 | –0.015 | –0.019 | –0.015 | –0.005 | –0.007 | –0.005 | –0.007 | –0.005 |
|  | [–0.021,  –0.013] | [–0.031,  –0.020] | [–0.021,  –0.013] | [–0.026,  –0.017] | [–0.021,  –0.013] | [–0.021,  –0.009] | [–0.033,  –0.012] | [–0.021,  –0.009] | [–0.027,  –0.011] | [–0.021,  –0.009] | [–0.009,  –0.002] | [–0.014,  0.001] | [–0.008,  –0.002] | [–0.011,  –0.002] | [–0.009,  –0.002] |
| Temporary × 2012 | –0.022 | –0.031 | –0.022 | –0.027 | –0.022 | –0.024 | –0.041 | –0.024 | –0.031 | –0.024 | –0.005 | –0.008 | –0.005 | –0.007 | –0.005 |
|  | [–0.026,  –0.018] | [–0.037,  –0.025] | [–0.026,  –0.018] | [–0.032,  –0.022] | [–0.026,  –0.018] | [–0.030,  –0.018] | [–0.050,  –0.031] | [–0.030,  –0.018] | [–0.039,  –0.024] | [–0.030,  –0.018] | [–0.009,  –0.001] | [–0.016,  –0.000] | [–0.009,  –0.001] | [–0.012,  –0.002] | [–0.009,  –0.001] |
| Temporary × 2015 | –0.024 | –0.036 | –0.024 | –0.030 | –0.025 | –0.023 | –0.038 | –0.023 | –0.031 | –0.024 | –0.008 | –0.017 | –0.008 | –0.011 | –0.009 |
|  | [–0.028,  –0.021] | [–0.041,  –0.030] | [–0.028,  –0.020] | [–0.035,  –0.025] | [–0.028,  –0.021] | [–0.030,  –0.017] | [–0.048,  –0.027] | [–0.030,  –0.017] | [–0.039,  –0.023] | [–0.030,  –0.017] | [–0.012,  –0.005] | [–0.023,  –0.011] | [–0.011,  –0.005] | [–0.015,  –0.007] | [–0.012,  –0.005] |
| Temporary × 2018 | –0.022 | –0.031 | –0.022 | –0.027 | –0.025 | –0.019 | –0.034 | –0.019 | –0.025 | –0.023 | –0.004 | –0.009 | –0.003 | –0.006 | –0.001 |
|  | [–0.026,  –0.019] | [–0.037,  –0.026] | [–0.026,  –0.018] | [–0.032,  –0.022] | [–0.029,  –0.020] | [–0.025,  –0.013] | [–0.044,  –0.024] | [–0.025,  –0.013] | [–0.033,  –0.017] | [–0.030,  –0.016] | [–0.007,  0.000] | [–0.017,  –0.002] | [–0.007,  0.000] | [–0.010,  –0.001] | [–0.006,  0.003] |
| Unemployed × 2000 | –0.015 | –0.024 | –0.016 | –0.019 | –0.015 | –0.005 | –0.011 | –0.006 | –0.008 | –0.005 | 0.003 | 0.003 | 0.003 | 0.004 | 0.003 |
|  | [–0.019,  –0.012] | [–0.029,  –0.019] | [–0.019,  –0.012] | [–0.023,  –0.015] | [–0.019,  –0.012] | [–0.012,  0.001] | [–0.021,  –0.000] | [–0.012,  0.001] | [–0.016,  0.001] | [–0.012,  0.001] | [–0.002,  0.007] | [–0.005,  0.011] | [–0.001,  0.008] | [–0.002,  0.009] | [–0.002,  0.007] |
| Unemployed × 2003 | –0.019 | –0.028 | –0.020 | –0.024 | –0.019 | –0.011 | –0.022 | –0.012 | –0.015 | –0.011 | –0.002 | –0.003 | –0.003 | –0.003 | –0.002 |
|  | [–0.022,  –0.016] | [–0.033,  –0.024] | [–0.023,  –0.016] | [–0.027,  –0.020] | [–0.022,  –0.016] | [–0.016,  –0.006] | [–0.030,  –0.015] | [–0.016,  –0.007] | [–0.021,  –0.009] | [–0.016,  –0.006] | [–0.004,  0.001] | [–0.008,  0.002] | [–0.005,  0.000] | [–0.006,  0.001] | [–0.004,  0.001] |
| Unemployed × 2006 | –0.023 | –0.034 | –0.024 | –0.029 | –0.023 | –0.015 | –0.028 | –0.015 | –0.021 | –0.016 | –0.003 | –0.006 | –0.003 | –0.004 | –0.003 |
|  | [–0.026,  –0.021] | [–0.038,  –0.031] | [–0.026,  –0.022] | [–0.032,  –0.026] | [–0.026,  –0.021] | [–0.019,  –0.012] | [–0.034,  –0.023] | [–0.019,  –0.012] | [–0.025,  –0.016] | [–0.019,  –0.012] | [–0.005,  –0.001] | [–0.010,  –0.003] | [–0.005,  –0.001] | [–0.006,  –0.001] | [–0.005,  –0.001] |
| Unemployed × 2009 | –0.023 | –0.035 | –0.024 | –0.029 | –0.024 | –0.020 | –0.036 | –0.020 | –0.026 | –0.020 | –0.004 | –0.008 | –0.004 | –0.006 | –0.004 |
|  | [–0.026,  –0.021] | [–0.038,  –0.031] | [–0.026,  –0.021] | [–0.032,  –0.026] | [–0.026,  –0.021] | [–0.023,  –0.016] | [–0.041,  –0.030] | [–0.024,  –0.017] | [–0.031,  –0.022] | [–0.023,  –0.016] | [–0.006,  –0.002] | [–0.012,  –0.004] | [–0.006,  –0.002] | [–0.008,  –0.003] | [–0.006,  –0.002] |
| Unemployed × 2012 | –0.027 | –0.038 | –0.028 | –0.033 | –0.027 | –0.022 | –0.037 | –0.023 | –0.029 | –0.023 | –0.002 | –0.004 | –0.002 | –0.003 | –0.002 |
|  | [–0.030,  –0.025] | [–0.041,  –0.034] | [–0.030,  –0.025] | [–0.036,  –0.030] | [–0.030,  –0.025] | [–0.026,  –0.019] | [–0.042,  –0.031] | [–0.027,  –0.020] | [–0.034,  –0.025] | [–0.026,  –0.019] | [–0.004,  0.001] | [–0.009,  –0.000] | [–0.004,  0.000] | [–0.006,  –0.000] | [–0.004,  0.001] |
| Unemployed × 2015 | –0.024 | –0.034 | –0.025 | –0.030 | –0.024 | –0.021 | –0.038 | –0.022 | –0.028 | –0.021 | –0.002 | –0.007 | –0.002 | –0.003 | –0.002 |
|  | [–0.027,  –0.022] | [–0.038,  –0.031] | [–0.027,  –0.022] | [–0.033,  –0.027] | [–0.027,  –0.022] | [–0.025,  –0.018] | [–0.044,  –0.031] | [–0.026,  –0.018] | [–0.033,  –0.024] | [–0.025,  –0.018] | [–0.004,  0.001] | [–0.011,  –0.002] | [–0.004,  0.001] | [–0.006,  –0.000] | [–0.004,  0.001] |
| Unemployed × 2018 | –0.023 | –0.032 | –0.023 | –0.028 | –0.024 | –0.018 | –0.031 | –0.018 | –0.024 | –0.021 | –0.001 | –0.005 | –0.001 | –0.002 | –0.001 |
|  | [–0.025,  –0.020] | [–0.036,  –0.028] | [–0.025,  –0.020] | [–0.031,  –0.024] | [–0.027,  –0.021] | [–0.022,  –0.014] | [–0.038,  –0.024] | [–0.022,  –0.014] | [–0.029,  –0.019] | [–0.026,  –0.016] | [–0.004,  0.001] | [–0.010,  –0.000] | [–0.004,  0.001] | [–0.005,  0.001] | [–0.004,  0.002] |
| Inactive × 2000 | 0.044 | 0.084 | 0.044 | 0.056 | 0.044 | 0.011 | 0.018 | 0.011 | 0.014 | 0.011 | 0.003 | 0.004 | 0.003 | 0.004 | 0.003 |
|  | [0.038,  0.050] | [0.074,  0.094] | [0.038,  0.050] | [0.049,  0.064] | [0.038,  0.050] | [0.006,  0.016] | [0.011,  0.026] | [0.006,  0.016] | [0.008,  0.020] | [0.006,  0.016] | [0.000,  0.006] | [–0.001,  0.009] | [0.000,  0.006] | [0.001,  0.007] | [0.000,  0.006] |
| Inactive × 2003 | 0.064 | 0.118 | 0.062 | 0.084 | 0.063 | 0.029 | 0.051 | 0.030 | 0.039 | 0.029 | 0.007 | 0.013 | 0.007 | 0.009 | 0.007 |
|  | [0.057,  0.070] | [0.107,  0.129] | [0.056,  0.069] | [0.076,  0.092] | [0.056,  0.069] | [0.025,  0.034] | [0.044,  0.059] | [0.025,  0.034] | [0.033,  0.044] | [0.025,  0.034] | [0.005,  0.009] | [0.009,  0.017] | [0.005,  0.009] | [0.006,  0.012] | [0.005,  0.009] |
| Inactive × 2006 | 0.085 | 0.152 | 0.083 | 0.112 | 0.084 | 0.033 | 0.060 | 0.033 | 0.043 | 0.033 | 0.007 | 0.012 | 0.007 | 0.008 | 0.007 |
|  | [0.079,  0.091] | [0.142,  0.162] | [0.078,  0.089] | [0.104,  0.120] | [0.078,  0.090] | [0.029,  0.037] | [0.054,  0.067] | [0.030,  0.037] | [0.039,  0.048] | [0.030,  0.037] | [0.005,  0.008] | [0.008,  0.015] | [0.005,  0.008] | [0.006,  0.010] | [0.005,  0.008] |
| Inactive × 2009 | 0.092 | 0.158 | 0.088 | 0.119 | 0.090 | 0.028 | 0.049 | 0.029 | 0.036 | 0.028 | 0.007 | 0.012 | 0.007 | 0.008 | 0.007 |
|  | [0.085,  0.098] | [0.147,  0.168] | [0.082,  0.095] | [0.110,  0.127] | [0.084,  0.096] | [0.025,  0.032] | [0.043,  0.055] | [0.025,  0.032] | [0.032,  0.041] | [0.025,  0.032] | [0.005,  0.009] | [0.009,  0.016] | [0.005,  0.009] | [0.006,  0.011] | [0.005,  0.009] |
| Inactive × 2012 | 0.103 | 0.179 | 0.100 | 0.134 | 0.101 | 0.028 | 0.052 | 0.029 | 0.038 | 0.028 | 0.011 | 0.020 | 0.012 | 0.014 | 0.012 |
|  | [0.095,  0.111] | [0.166,  0.192] | [0.092,  0.107] | [0.124,  0.144] | [0.093,  0.108] | [0.024,  0.033] | [0.044,  0.059] | [0.024,  0.033] | [0.032,  0.044] | [0.024,  0.033] | [0.009,  0.014] | [0.016,  0.024] | [0.010,  0.014] | [0.011,  0.017] | [0.010,  0.014] |
| Inactive × 2015 | 0.089 | 0.151 | 0.087 | 0.113 | 0.088 | 0.030 | 0.049 | 0.030 | 0.037 | 0.030 | 0.012 | 0.019 | 0.012 | 0.015 | 0.012 |
|  | [0.081,  0.097] | [0.137,  0.164] | [0.079,  0.095] | [0.103,  0.123] | [0.080,  0.095] | [0.025,  0.034] | [0.041,  0.057] | [0.026,  0.035] | [0.031,  0.043] | [0.025,  0.034] | [0.010,  0.014] | [0.015,  0.024] | [0.010,  0.014] | [0.012,  0.017] | [0.010,  0.014] |
| Inactive × 2018 | 0.108 | 0.184 | 0.107 | 0.137 | 0.111 | 0.039 | 0.068 | 0.040 | 0.051 | 0.039 | 0.012 | 0.020 | 0.012 | 0.015 | 0.013 |
|  | [0.099,  0.118] | [0.169,  0.199] | [0.098,  0.115] | [0.125,  0.148] | [0.100,  0.121] | [0.034,  0.044] | [0.059,  0.076] | [0.035,  0.044] | [0.044,  0.057] | [0.033,  0.045] | [0.010,  0.014] | [0.016,  0.025] | [0.010,  0.014] | [0.012,  0.018] | [0.010,  0.015] |
| Observations | 1569714 | 1043704 | 1549171 | 1280440 | 1504401 | 1005919 | 558347 | 990800 | 772975 | 966535 | 1088144 | 474249 | 1081349 | 818502 | 1042702 |

Source: LFS, Italy 2000–2020

# **Table S2** AMEs from logistic regression models examining first, second, and higher childbirths as a function of employment situation (1-year lagged). 95% confidence intervals in brackets.

**Men**

|  | M0 | M1 | M2 | M0 | M1 | M2 | M0 | M1 | M2 |
| --- | --- | --- | --- | --- | --- | --- | --- | --- | --- |
|  | 1st Birth | 1st Birth | 1st Birth | 2nd Birth | 2nd Birth | 2nd Birth | 3rd Birth | 3rd Birth | 3rd Birth |
| Self-empl × 2000 | 0.002 | 0.002 | 0.003 | 0.005 | 0.006 | 0.003 | 0.018 | 0.018 | 0.003 |
|  | [–0.014,0.018] | [–0.013,0.018] | [–0.013,0.020] | [–0.025,0.036] | [–0.025,0.036] | [–0.013,0.020] | [–0.005,0.040] | [–0.005,0.040] | [–0.013,0.020] |
| Self-empl × 2003 | –0.012 | –0.012 | –0.012 | 0.009 | 0.008 | –0.012 | 0.004 | 0.004 | –0.012 |
|  | [–0.026,0.001] | [–0.025,0.002] | [–0.025,0.002] | [–0.024,0.042] | [–0.024,0.041] | [–0.025,0.002] | [–0.014,0.022] | [–0.014,0.022] | [–0.025,0.002] |
| Self-empl × 2006 | –0.015 | –0.014 | –0.014 | –0.001 | –0.002 | –0.014 | –0.001 | –0.001 | –0.014 |
|  | [–0.024,–0.006] | [–0.023,–0.005] | [–0.023,–0.005] | [–0.022,0.020] | [–0.022,0.019] | [–0.023,–0.005] | [–0.013,0.011] | [–0.013,0.010] | [–0.023,–0.005] |
| Self-empl × 2009 | –0.021 | –0.021 | –0.021 | 0.002 | 0.002 | –0.021 | 0.015 | 0.014 | –0.021 |
|  | [–0.031,–0.012] | [–0.030,–0.011] | [–0.030,–0.011] | [–0.024,0.027] | [–0.023,0.028] | [–0.030,–0.011] | [–0.002,0.032] | [–0.003,0.031] | [–0.030,–0.011] |
| Self-empl × 2012 | –0.007 | –0.007 | –0.007 | –0.013 | –0.013 | –0.007 | –0.008 | –0.008 | –0.007 |
|  | [–0.024,0.010] | [–0.024,0.010] | [–0.024,0.010] | [–0.045,0.018] | [–0.045,0.018] | [–0.024,0.010] | [–0.024,0.009] | [–0.024,0.009] | [–0.024,0.010] |
| Self-empl × 2015 | –0.010 | –0.010 | –0.010 | –0.011 | –0.011 | –0.010 | 0.036 | 0.036 | –0.010 |
|  | [–0.024,0.004] | [–0.024,0.004] | [–0.024,0.004] | [–0.041,0.018] | [–0.041,0.019] | [–0.024,0.004] | [0.007,0.066] | [0.007,0.065] | [–0.024,0.004] |
| Self-empl × 2018 | –0.012 | –0.012 | –0.012 | –0.024 | –0.024 | –0.012 | 0.008 | 0.008 | –0.012 |
|  | [–0.027,0.003] | [–0.027,0.003] | [–0.027,0.003] | [–0.055,0.007] | [–0.055,0.007] | [–0.027,0.003] | [–0.015,0.031] | [–0.015,0.031] | [–0.027,0.003] |
| Temporary × 2000 | –0.011 | –0.010 | –0.011 | –0.003 | –0.003 | –0.011 | –0.001 | –0.002 | –0.011 |
|  | [–0.018,–0.003] | [–0.018,–0.003] | [–0.018,–0.004] | [–0.020,0.014] | [–0.020,0.015] | [–0.018,–0.004] | [–0.010,0.007] | [–0.010,0.007] | [–0.018,–0.004] |
| Temporary × 2003 | –0.010 | –0.009 | –0.009 | –0.006 | –0.005 | –0.009 | 0.004 | 0.003 | –0.009 |
|  | [–0.016,–0.004] | [–0.015,–0.003] | [–0.015,–0.004] | [–0.019,0.008] | [–0.018,0.009] | [–0.015,–0.004] | [–0.003,0.012] | [–0.004,0.011] | [–0.015,–0.004] |
| Temporary × 2006 | –0.019 | –0.019 | –0.019 | –0.003 | –0.002 | –0.019 | –0.004 | –0.005 | –0.019 |
|  | [–0.023,–0.016] | [–0.022,–0.015] | [–0.023,–0.015] | [–0.012,0.007] | [–0.012,0.008] | [–0.023,–0.015] | [–0.009,0.000] | [–0.010,–0.001] | [–0.023,–0.015] |
| Temporary × 2009 | –0.015 | –0.015 | –0.015 | –0.010 | –0.009 | –0.015 | 0.006 | 0.005 | –0.015 |
|  | [–0.020,–0.011] | [–0.019,–0.011] | [–0.019,–0.011] | [–0.020,–0.001] | [–0.019,–0.000] | [–0.019,–0.011] | [–0.000,0.012] | [–0.001,0.011] | [–0.019,–0.011] |
| Temporary × 2012 | –0.013 | –0.013 | –0.013 | –0.009 | –0.008 | –0.013 | –0.001 | –0.001 | –0.013 |
|  | [–0.018,–0.009] | [–0.017,–0.009] | [–0.017,–0.009] | [–0.019,0.000] | [–0.018,0.002] | [–0.017,–0.009] | [–0.006,0.005] | [–0.007,0.004] | [–0.017,–0.009] |
| Temporary × 2015 | –0.016 | –0.015 | –0.015 | –0.010 | –0.009 | –0.015 | –0.001 | –0.002 | –0.015 |
|  | [–0.020,–0.012] | [–0.019,–0.011] | [–0.019,–0.011] | [–0.020,–0.001] | [–0.019,0.001] | [–0.019,–0.011] | [–0.006,0.005] | [–0.007,0.004] | [–0.019,–0.011] |
| Temporary × 2018 | –0.015 | –0.014 | –0.014 | –0.011 | –0.010 | –0.014 | 0.006 | 0.005 | –0.014 |
|  | [–0.018,–0.011] | [–0.018,–0.010] | [–0.018,–0.010] | [–0.020,–0.001] | [–0.019,–0.000] | [–0.018,–0.010] | [–0.000,0.013] | [–0.001,0.012] | [–0.018,–0.010] |
| Age, age-squared, education | Yes | Yes | Yes | Yes | Yes | Yes | Yes | Yes | Yes |
| Social Class (ESeG) | No | Yes | Yes | No | Yes | Yes | No | Yes | Yes |
| Regional unemployment (squared) | No | No | Yes | No | No | Yes | No | No | Yes |
| Observations | 827496 | 827496 | 825657 | 516630 | 516630 | 825657 | 529172 | 529172 | 825657 |

(continues of the next page)

(continues from the previous page)

**Women**

|  | M0 | M1 | M2 | M0 | M1 | M2 | M0 | M1 | M2 |
| --- | --- | --- | --- | --- | --- | --- | --- | --- | --- |
|  | 1st Birth | 1st Birth | 1st Birth | 2nd Birth | 2nd Birth | 2nd Birth | 3rd Birth | 3rd Birth | 3rd Birth |
| Self-empl × 2000 | –0.017 | –0.016 | –0.016 | –0.018 | –0.018 | –0.016 | 0.000 | 0.000 | –0.016 |
|  | [–0.030,–0.003] | [–0.030,–0.003] | [–0.030,–0.001] | [–0.039,0.003] | [–0.038,0.003] | [–0.030,–0.001] | [0.000,0.000] | [0.000,0.000] | [–0.030,–0.001] |
| Self-empl × 2003 | –0.013 | –0.013 | –0.013 | –0.012 | –0.012 | –0.013 | 0.002 | 0.001 | –0.013 |
|  | [–0.029,0.003] | [–0.029,0.003] | [–0.029,0.003] | [–0.036,0.012] | [–0.036,0.012] | [–0.029,0.003] | [–0.014,0.017] | [–0.014,0.017] | [–0.029,0.003] |
| Self-empl × 2006 | –0.023 | –0.023 | –0.023 | –0.022 | –0.022 | –0.023 | 0.004 | 0.004 | –0.023 |
|  | [–0.031,–0.014] | [–0.031,–0.014] | [–0.031,–0.015] | [–0.035,–0.009] | [–0.035,–0.009] | [–0.031,–0.015] | [–0.007,0.015] | [–0.007,0.015] | [–0.031,–0.015] |
| Self-empl × 2009 | –0.018 | –0.019 | –0.019 | –0.015 | –0.015 | –0.019 | 0.008 | 0.008 | –0.019 |
|  | [–0.029,–0.007] | [–0.030,–0.008] | [–0.030,–0.008] | [–0.033,0.004] | [–0.033,0.004] | [–0.030,–0.008] | [–0.007,0.024] | [–0.007,0.023] | [–0.030,–0.008] |
| Self-empl × 2012 | –0.022 | –0.022 | –0.022 | –0.036 | –0.037 | –0.022 | 0.004 | 0.004 | –0.022 |
|  | [–0.037,–0.006] | [–0.037,–0.006] | [–0.037,–0.006] | [–0.054,–0.019] | [–0.055,–0.019] | [–0.037,–0.006] | [–0.018,0.026] | [–0.018,0.025] | [–0.037,–0.006] |
| Self-empl × 2015 | –0.024 | –0.025 | –0.025 | –0.039 | –0.039 | –0.025 | 0.000 | 0.000 | –0.025 |
|  | [–0.036,–0.013] | [–0.037,–0.013] | [–0.037,–0.013] | [–0.053,–0.025] | [–0.053,–0.026] | [–0.037,–0.013] | [–0.016,0.016] | [–0.016,0.016] | [–0.037,–0.013] |
| Self-empl × 2018 | –0.026 | –0.027 | –0.026 | –0.032 | –0.033 | –0.026 | –0.005 | –0.005 | –0.026 |
|  | [–0.037,–0.015] | [–0.038,–0.015] | [–0.038,–0.015] | [–0.049,–0.015] | [–0.050,–0.016] | [–0.038,–0.015] | [–0.017,0.006] | [–0.016,0.006] | [–0.038,–0.015] |
| Temporary × 2000 | –0.008 | –0.008 | –0.007 | 0.011 | 0.011 | –0.007 | –0.003 | –0.003 | –0.007 |
|  | [–0.015,–0.000] | [–0.015,–0.000] | [–0.015,0.000] | [–0.004,0.026] | [–0.004,0.026] | [–0.015,0.000] | [–0.009,0.003] | [–0.009,0.003] | [–0.015,0.000] |
| Temporary × 2003 | –0.007 | –0.007 | –0.007 | –0.002 | –0.001 | –0.007 | 0.002 | 0.002 | –0.007 |
|  | [–0.013,–0.001] | [–0.013,–0.001] | [–0.013,–0.001] | [–0.011,0.007] | [–0.010,0.008] | [–0.013,–0.001] | [–0.002,0.007] | [–0.002,0.006] | [–0.013,–0.001] |
| Temporary × 2006 | –0.015 | –0.016 | –0.016 | –0.007 | –0.007 | –0.016 | 0.001 | 0.000 | –0.016 |
|  | [–0.019,–0.012] | [–0.019,–0.012] | [–0.020,–0.012] | [–0.013,–0.001] | [–0.013,–0.001] | [–0.020,–0.012] | [–0.003,0.004] | [–0.003,0.004] | [–0.020,–0.012] |
| Temporary × 2009 | –0.009 | –0.009 | –0.009 | –0.007 | –0.007 | –0.009 | –0.003 | –0.003 | –0.009 |
|  | [–0.013,–0.005] | [–0.014,–0.005] | [–0.014,–0.005] | [–0.013,–0.000] | [–0.013,–0.000] | [–0.014,–0.005] | [–0.006,0.001] | [–0.006,0.000] | [–0.014,–0.005] |
| Temporary × 2012 | –0.015 | –0.015 | –0.015 | –0.018 | –0.019 | –0.015 | –0.003 | –0.003 | –0.015 |
|  | [–0.019,–0.011] | [–0.020,–0.011] | [–0.020,–0.011] | [–0.025,–0.012] | [–0.025,–0.012] | [–0.020,–0.011] | [–0.006,0.001] | [–0.006,0.001] | [–0.020,–0.011] |
| Temporary × 2015 | –0.017 | –0.018 | –0.017 | –0.016 | –0.016 | –0.017 | –0.006 | –0.006 | –0.017 |
|  | [–0.022,–0.013] | [–0.022,–0.013] | [–0.022,–0.013] | [–0.022,–0.009] | [–0.023,–0.010] | [–0.022,–0.013] | [–0.009,–0.003] | [–0.009,–0.003] | [–0.022,–0.013] |
| Temporary × 2018 | –0.015 | –0.015 | –0.015 | –0.012 | –0.013 | –0.015 | –0.001 | –0.001 | –0.015 |
|  | [–0.019,–0.010] | [–0.019,–0.011] | [–0.019,–0.010] | [–0.019,–0.006] | [–0.019,–0.006] | [–0.019,–0.010] | [–0.005,0.003] | [–0.005,0.002] | [–0.019,–0.010] |
| Age, age-squared, education | Yes | Yes | Yes | Yes | Yes | Yes | Yes | Yes | Yes |
| Social Class (ESeG) | No | Yes | Yes | No | Yes | Yes | No | Yes | Yes |
| Regional unemployment (squared) | No | No | Yes | No | No | Yes | No | No | Yes |
| Observations | 827496 | 827496 | 825657 | 516630 | 516630 | 825657 | 529172 | 529172 | 825657 |

Source: LFS, Italy 2000–2020

# **Table S3** AMEs from logistic regression models examining first, second, and higher childbirths as a function of employment situation (1-year lagged). 95% confidence intervals in brackets.

**Men**

|  | M0 | M1 | M2 | M3 | M0 | M1 | M2 | M3 | M0 | M1 | M2 | M3 |
| --- | --- | --- | --- | --- | --- | --- | --- | --- | --- | --- | --- | --- |
|  | 1st Birth | 1st Birth | 1st Birth | 1st Birth | 2nd Birth | 2nd Birth | 2nd Birth | 2nd Birth | 3rd Birth | 3rd Birth | 3rd Birth | 3rd Birth |
| Self-empl × 2009 | –0.021 | –0.021 | –0.020 | –0.020 | 0.002 | 0.003 | 0.004 | 0.004 | 0.014 | 0.014 | 0.014 | 0.013 |
|  | [–0.031,  –0.012] | [–0.030,  –0.011] | [–0.029,  –0.010] | [–0.029,  –0.010] | [–0.023,  0.027] | [–0.022,  0.028] | [–0.021,  0.028] | [–0.021,  0.029] | [–0.002,  0.031] | [–0.003,  0.031] | [–0.003,  0.031] | [–0.003,  0.029] |
| Self-empl × 2012 | –0.007 | –0.007 | –0.005 | –0.005 | –0.013 | –0.013 | –0.011 | –0.011 | –0.007 | –0.008 | –0.008 | –0.009 |
|  | [–0.024,  0.010] | [–0.024,  0.010] | [–0.022,  0.012] | [–0.022,  0.012] | [–0.044,  0.018] | [–0.044,  0.018] | [–0.042,  0.020] | [–0.042,  0.020] | [–0.023,  0.008] | [–0.024,  0.008] | [–0.024,  0.008] | [–0.024,  0.007] |
| Self-empl × 2015 | –0.010 | –0.010 | –0.008 | –0.008 | –0.011 | –0.010 | –0.008 | –0.008 | 0.035 | 0.035 | 0.035 | 0.037 |
|  | [–0.024,  0.004] | [–0.024,  0.004] | [–0.022,  0.006] | [–0.022,  0.006] | [–0.040,  0.019] | [–0.040,  0.019] | [–0.038,  0.022] | [–0.038,  0.021] | [0.007,  0.064] | [0.006,  0.064] | [0.006,  0.063] | [0.006,  0.067] |
| Self-empl × 2018 | –0.012 | –0.012 | –0.010 | –0.010 | –0.024 | –0.023 | –0.022 | –0.022 | 0.008 | 0.008 | 0.008 | 0.007 |
|  | [–0.027,  0.002] | [–0.027,  0.003] | [–0.025,  0.005] | [–0.025,  0.005] | [–0.054,  0.007] | [–0.054,  0.007] | [–0.053,  0.008] | [–0.052,  0.008] | [–0.015,  0.031] | [–0.015,  0.031] | [–0.015,  0.031] | [–0.016,  0.030] |
| Temporary × 2009 | –0.016 | –0.015 | –0.013 | –0.013 | –0.009 | –0.009 | –0.006 | –0.006 | 0.005 | 0.004 | 0.004 | 0.005 |
|  | [–0.020,  –0.012] | [–0.019,  –0.011] | [–0.017,  –0.009] | [–0.017,  –0.009] | [–0.019,  0.000] | [–0.018,  0.001] | [–0.016,  0.003] | [–0.016,  0.003] | [–0.000,  0.011] | [–0.001,  0.010] | [–0.001,  0.010] | [–0.001,  0.010] |
| Temporary × 2012 | –0.013 | –0.013 | –0.010 | –0.010 | –0.008 | –0.007 | –0.005 | –0.005 | –0.001 | –0.002 | –0.002 | –0.002 |
|  | [–0.018,  –0.009] | [–0.017,  –0.009] | [–0.015,  –0.006] | [–0.015,  –0.006] | [–0.018,  0.001] | [–0.017,  0.002] | [–0.015,  0.005] | [–0.015,  0.004] | [–0.006,  0.005] | [–0.007,  0.004] | [–0.007,  0.004] | [–0.007,  0.004] |
| Temporary × 2015 | –0.016 | –0.015 | –0.013 | –0.013 | –0.009 | –0.008 | –0.006 | –0.007 | –0.001 | –0.002 | –0.002 | –0.002 |
|  | [–0.020,  –0.012] | [–0.019,  –0.011] | [–0.017,  –0.009] | [–0.017,  –0.009] | [–0.019,  0.000] | [–0.018,  0.001] | [–0.016,  0.004] | [–0.016,  0.003] | [–0.006,  0.004] | [–0.007,  0.003] | [–0.007,  0.003] | [–0.008,  0.004] |
| Temporary × 2018 | –0.015 | –0.014 | –0.012 | –0.012 | –0.010 | –0.009 | –0.007 | –0.007 | 0.006 | 0.005 | 0.005 | 0.005 |
|  | [–0.018,  –0.011] | [–0.018,  –0.010] | [–0.016,  –0.008] | [–0.016,  –0.008] | [–0.019,  –0.000] | [–0.019,  0.001] | [–0.016,  0.003] | [–0.017,  0.003] | [–0.000,  0.012] | [–0.001,  0.011] | [–0.001,  0.011] | [–0.001,  0.011] |
| Age, age-squared, education | Yes | Yes | Yes | Yes | Yes | Yes | Yes | Yes | Yes | Yes | Yes | Yes |
| Social Class (ESeG) | No | Yes | Yes | Yes | No | Yes | Yes | Yes | No | Yes | Yes | Yes |
| Net salary | No | No | Yes | Yes | No | No | Yes | Yes | No | No | Yes | Yes |
| Regional unemployment (squared) | No | No | No | Yes | No | No | No | Yes | No | No | No | Yes |
| Observations | 504734 | 504734 | 504734 | 504734 | 321495 | 321495 | 321495 | 321495 | 344202 | 344202 | 344202 | 344202 |

(continues of the next page)

(continues from the previous page)

**Women**

|  | M0 | M1 | M2 | M3 | M0 | M1 | M2 | M3 | M0 | M1 | M2 | M3 |
| --- | --- | --- | --- | --- | --- | --- | --- | --- | --- | --- | --- | --- |
|  | 1st Birth | 1st Birth | 1st Birth | 1st Birth | 2nd Birth | 2nd Birth | 2nd Birth | 2nd Birth | 3rd Birth | 3rd Birth | 3rd Birth | 3rd Birth |
| Self-empl × 2009 | –0.018 | –0.019 | –0.018 | –0.019 | –0.014 | –0.015 | –0.015 | –0.015 | 0.008 | 0.007 | 0.007 | 0.006 |
|  | [–0.029,  –0.007] | [–0.030,  –0.008] | [–0.030,  –0.007] | [–0.031,  –0.007] | [–0.033,  0.004] | [–0.033,  0.004] | [–0.033,  0.004] | [–0.034,  0.004] | [–0.007,  0.023] | [–0.007,  0.022] | [–0.007,  0.022] | [–0.007,  0.019] |
| Self-empl × 2012 | –0.022 | –0.022 | –0.022 | –0.022 | –0.036 | –0.036 | –0.037 | –0.037 | 0.004 | 0.004 | 0.004 | 0.004 |
|  | [–0.037,  –0.006] | [–0.037,  –0.006] | [–0.038,  –0.005] | [–0.038,  –0.005] | [–0.054,  –0.018] | [–0.054,  –0.018] | [–0.055,  –0.019] | [–0.055,  –0.019] | [–0.018,  0.026] | [–0.018,  0.026] | [–0.018,  0.025] | [–0.018,  0.025] |
| Self-empl × 2015 | –0.024 | –0.025 | –0.025 | –0.025 | –0.038 | –0.039 | –0.039 | –0.039 | 0.000 | 0.000 | 0.000 | –0.000 |
|  | [–0.036,  –0.013] | [–0.037,  –0.013] | [–0.038,  –0.012] | [–0.037,  –0.012] | [–0.052,  –0.025] | [–0.052,  –0.025] | [–0.054,  –0.025] | [–0.053,  –0.025] | [–0.016,  0.017] | [–0.016,  0.016] | [–0.015,  0.016] | [–0.016,  0.016] |
| Self-empl × 2018 | –0.026 | –0.026 | –0.027 | –0.027 | –0.032 | –0.032 | –0.033 | –0.033 | –0.005 | –0.005 | –0.005 | –0.005 |
|  | [–0.037,  –0.015] | [–0.038,  –0.015] | [–0.039,  –0.015] | [–0.039,  –0.015] | [–0.049,  –0.015] | [–0.049,  –0.015] | [–0.051,  –0.016] | [–0.051,  –0.016] | [–0.017,  0.006] | [–0.016,  0.006] | [–0.016,  0.006] | [–0.016,  0.006] |
| Temporary × 2009 | –0.009 | –0.009 | –0.008 | –0.008 | –0.006 | –0.006 | –0.006 | –0.006 | –0.003 | –0.003 | –0.003 | –0.002 |
|  | [–0.013,  –0.005] | [–0.014,  –0.005] | [–0.013,  –0.004] | [–0.013,  –0.004] | [–0.013,  0.000] | [–0.013,  0.000] | [–0.013,  0.000] | [–0.013,  0.000] | [–0.006,  0.000] | [–0.006,  0.000] | [–0.006,  0.000] | [–0.005,  0.000] |
| Temporary × 2012 | –0.015 | –0.015 | –0.015 | –0.015 | –0.018 | –0.018 | –0.018 | –0.018 | –0.003 | –0.003 | –0.003 | –0.003 |
|  | [–0.019,  –0.011] | [–0.020,  –0.011] | [–0.019,  –0.010] | [–0.019,  –0.010] | [–0.024,  –0.012] | [–0.024,  –0.012] | [–0.025,  –0.012] | [–0.025,  –0.012] | [–0.006,  0.001] | [–0.006,  0.001] | [–0.006,  0.000] | [–0.006,  0.000] |
| Temporary × 2015 | –0.017 | –0.017 | –0.017 | –0.017 | –0.015 | –0.016 | –0.016 | –0.016 | –0.006 | –0.006 | –0.006 | –0.006 |
|  | [–0.021,  –0.013] | [–0.022,  –0.013] | [–0.022,  –0.013] | [–0.022,  –0.013] | [–0.022,  –0.009] | [–0.022,  –0.009] | [–0.022,  –0.009] | [–0.022,  –0.009] | [–0.009,  –0.003] | [–0.008,  –0.003] | [–0.008,  –0.003] | [–0.009,  –0.003] |
| Temporary × 2018 | –0.014 | –0.015 | –0.014 | –0.014 | –0.012 | –0.012 | –0.012 | –0.013 | –0.001 | –0.001 | –0.001 | –0.001 |
|  | [–0.018,  –0.010] | [–0.019,  –0.010] | [–0.019,  –0.010] | [–0.019,  –0.010] | [–0.018,  –0.005] | [–0.019,  –0.006] | [–0.019,  –0.006] | [–0.019,  –0.006] | [–0.005,  0.002] | [–0.005,  0.002] | [–0.005,  0.002] | [–0.005,  0.002] |
| Age, age-squared, education | Yes | Yes | Yes | Yes | Yes | Yes | Yes | Yes | Yes | Yes | Yes | Yes |
| Social Class (ESeG) | No | Yes | Yes | Yes | No | Yes | Yes | Yes | No | Yes | Yes | Yes |
| Net salary | No | No | Yes | Yes | No | No | Yes | Yes | No | No | Yes | Yes |
| Regional unemployment (squared) | No | No | No | Yes | No | No | No | Yes | No | No | No | Yes |
| Observations | 504734 | 504734 | 504734 | 504734 | 321495 | 321495 | 321495 | 321495 | 344202 | 344202 | 344202 | 344202 |

Source: LFS, Italy 2000–2020

# **Table S4** AMEs from logistic regression models examining first, second, and higher childbirths as a function of employment situation (1-year lagged). 95% confidence intervals in brackets.

**Men**

|  | M0 | M1 | M2 | M3 | M4 | M0 | M1 | M2 | M3 | M4 | M0 | M1 | M2 | M3 | M4 |
| --- | --- | --- | --- | --- | --- | --- | --- | --- | --- | --- | --- | --- | --- | --- | --- |
|  | 1st Birth | 1st Birth | 1st Birth | 1st Birth | 1st Birth | 2nd Birth | 2nd Birth | 2nd Birth | 2nd Birth | 2nd Birth | 3rd Birth | 3rd Birth | 3rd Birth | 3rd Birth | 3rd Birth |
| Self-empl × 2009 | –0.051 | –0.051 | –0.051 | –0.052 | –0.054 | 0.007 | 0.008 | 0.008 | 0.008 | 0.008 | 0.017 | 0.017 | 0.017 | 0.017 | 0.014 |
|  | [–0.092, –0.010] | [–0.092, –0.009] | [–0.092, –0.010] | [–0.092, –0.011] | [–0.096, –0.011] | [–0.023,  0.038] | [–0.023,  0.038] | [–0.022,  0.038] | [–0.022,  0.038] | [–0.023,  0.038] | [–0.001,  0.034] | [–0.001,  0.034] | [–0.001,  0.035] | [–0.001,  0.034] | [–0.001,  0.030] |
| Self-empl × 2012 | –0.023 | –0.022 | –0.023 | –0.023 | –0.021 | –0.009 | –0.009 | –0.008 | –0.011 | –0.011 | –0.007 | –0.007 | –0.008 | –0.006 | –0.007 |
|  | [–0.081, 0.035] | [–0.080, 0.036] | [–0.080, 0.035] | [–0.080,  0.034] | [–0.078,  0.036] | [–0.048,  0.030] | [–0.048,  0.029] | [–0.047,  0.031] | [–0.048,  0.027] | [–0.048,  0.027] | [–0.024,  0.009] | [–0.024,  0.009] | [–0.024,  0.009] | [–0.022,  0.010] | [–0.023,  0.009] |
| Self-empl × 2015 | –0.024 | –0.024 | –0.025 | –0.025 | –0.022 | –0.008 | –0.007 | –0.006 | –0.007 | –0.008 | 0.035 | 0.034 | 0.034 | 0.034 | 0.037 |
|  | [–0.076, 0.029] | [–0.076, 0.028] | [–0.077,  0.026] | [–0.076,  0.026] | [–0.071,  0.028] | [–0.044,  0.029] | [–0.044,  0.029] | [–0.043,  0.031] | [–0.044,  0.029] | [–0.044,  0.029] | [0.005,  0.064] | [0.005,  0.064] | [0.005,  0.064] | [0.006,  0.063] | [0.006,  0.067] |
| Self-empl × 2018 | –0.042 | –0.042 | –0.043 | –0.043 | –0.043 | –0.019 | –0.018 | –0.018 | –0.019 | –0.018 | 0.004 | 0.004 | 0.004 | 0.004 | 0.004 |
|  | [–0.093,  0.009] | [–0.092, 0.009] | [–0.093,  0.007] | [–0.092,  0.007] | [–0.092,  0.007] | [–0.059,  0.021] | [–0.058,  0.022] | [–0.058,  0.021] | [–0.058,  0.020] | [–0.058,  0.021] | [–0.018,  0.027] | [–0.018,  0.027] | [–0.018,  0.027] | [–0.017,  0.026] | [–0.018,  0.025] |
| Temporary × 2009 | –0.022 | –0.022 | –0.021 | –0.023 | –0.024 | 0.001 | 0.002 | 0.003 | 0.002 | 0.003 | 0.008 | 0.007 | 0.007 | 0.005 | 0.006 |
|  | [–0.039, –0.004] | [–0.039,  –0.004] | [–0.038,  –0.004] | [–0.040,  –0.006] | [–0.042,  –0.007] | [–0.012,  0.014] | [–0.011,  0.015] | [–0.010,  0.016] | [–0.011,  0.015] | [–0.010,  0.016] | [0.001,  0.014] | [0.001,  0.013] | [0.000,  0.013] | [–0.000,  0.011] | [0.000,  0.011] |
| Temporary × 2012 | –0.016 | –0.015 | –0.014 | –0.017 | –0.017 | –0.002 | –0.001 | 0.001 | –0.001 | –0.001 | –0.000 | –0.001 | –0.001 | –0.003 | –0.002 |
|  | [–0.034,  0.003] | [–0.033,  0.004] | [–0.032,  0.005] | [–0.035,  0.001] | [–0.035,  0.001] | [–0.014,  0.011] | [–0.013,  0.012] | [–0.012,  0.013] | [–0.013,  0.011] | [–0.014,  0.011] | [–0.006,  0.006] | [–0.007,  0.005] | [–0.007,  0.005] | [–0.008,  0.003] | [–0.008,  0.003] |
| Temporary × 2015 | –0.024 | –0.024 | –0.023 | –0.025 | –0.024 | –0.000 | 0.001 | 0.002 | 0.001 | –0.000 | 0.001 | 0.000 | 0.000 | –0.001 | –0.000 |
|  | [–0.043,  –0.006] | [–0.042,  –0.006] | [–0.041,  –0.005] | [–0.043,  –0.007] | [–0.041,  –0.007] | [–0.013,  0.012] | [–0.012,  0.014] | [–0.010,  0.015] | [–0.012,  0.014] | [–0.013,  0.012] | [–0.005,  0.007] | [–0.005,  0.006] | [–0.006,  0.006] | [–0.006,  0.004] | [–0.006,  0.006] |
| Temporary × 2018 | –0.001 | 0.000 | 0.001 | –0.002 | –0.004 | –0.001 | 0.000 | 0.002 | 0.000 | –0.000 | 0.010 | 0.009 | 0.009 | 0.0306 | 0.007 |
|  | [–0.019,  0.017] | [–0.018,  0.018] | [–0.018,  0.019] | [–0.020,  0.016] | [–0.022,  0.013] | [–0.014,  0.011] | [–0.013,  0.013] | [–0.011,  0.015] | [–0.012,  0.013] | [–0.013,  0.012] | [0.003,  0.017] | [0.002,  0.015] | [0.002,  0.015] | [0.000,  0.013] | [0.001,  0.014] |
| Age, age-squared, education | Yes | Yes | Yes | Yes | Yes | Yes | Yes | Yes | Yes | Yes | Yes | Yes | Yes | Yes | Yes |
| Social Class (ESeG) | No | Yes | Yes | Yes | Yes | No | Yes | Yes | Yes | Yes | No | Yes | Yes | Yes | Yes |
| Net salary | No | No | Yes | No | Yes | No | No | Yes | No | Yes | No | No | Yes | No | Yes |
| Partner’s Social Class (ESeG) | No | No | No | Yes | Yes | No | No | No | Yes | Yes | No | No | No | Yes | Yes |
| Couple net salary | No | No | No | Yes | Yes | No | No | No | Yes | Yes | No | No | No | Yes | Yes |
| Regional unemployment (squared) | No | No | No | No | Yes | No | No | No | No | Yes | No | No | No | No | Yes |
| Observations | 160230 | 160230 | 160230 | 160230 | 160230 | 258171 | 258171 | 258171 | 258171 | 258171 | 320285 | 320285 | 320285 | 320285 | 320285 |

(continues on the next page)

(continues from the previous page)

**Women**

|  | M0 | M1 | M2 | M3 | M4 | M0 | M1 | M2 | M3 | M4 | M0 | M1 | M2 | M3 | M4 |
| --- | --- | --- | --- | --- | --- | --- | --- | --- | --- | --- | --- | --- | --- | --- | --- |
|  | 1st Birth | 1st Birth | 1st Birth | 1st Birth | 1st Birth | 2nd Birth | 2nd Birth | 2nd Birth | 2nd Birth | 2nd Birth | 3rd Birth | 3rd Birth | 3rd Birth | 3rd Birth | 3rd Birth |
| Self-empl × 2009 | –0.033 | –0.034 | –0.034 | –0.036 | –0.037 | –0.011 | –0.012 | –0.012 | –0.013 | –0.013 | 0.010 | 0.010 | 0.009 | 0.010 | 0.008 |
|  | [–0.064,  –0.002] | [–0.064,  –0.003] | [–0.064,  –0.003] | [–0.066,  –0.005] | [–0.069,  –0.005] | [–0.037,  0.014] | [–0.037,  0.013] | [–0.038,  0.014] | [–0.038,  0.012] | [–0.038,  0.013] | [–0.007,  0.027] | [–0.007,  0.026] | [–0.007,  0.025] | [–0.007,  0.027] | [–0.007,  0.023] |
| Self-empl × 2012 | –0.047 | –0.048 | –0.048 | –0.049 | –0.049 | –0.039 | –0.039 | –0.040 | –0.040 | –0.040 | 0.010 | 0.010 | 0.009 | 0.009 | 0.010 |
|  | [–0.086,  –0.008] | [–0.087,  –0.009] | [–0.087,  –0.009] | [–0.088,  –0.010] | [–0.088,  –0.010] | [–0.065,  –0.013] | [–0.065,  –0.013] | [–0.066,  –0.013] | [–0.066,  –0.013] | [–0.066,  –0.013] | [–0.019, 0.039] | [–0.019,  0.039] | [–0.019,  0.038] | [–0.021,  0.039] | [–0.021,  0.041] |
| Self-empl × 2015 | –0.055 | –0.056 | –0.056 | –0.058 | –0.055 | –0.045 | –0.045 | –0.046 | –0.046 | –0.045 | 0.004 | 0.003 | 0.003 | 0.003 | 0.003 |
|  | [–0.086,  –0.023] | [–0.087,  –0.025] | [–0.087,  –0.026] | [–0.088,  –0.028] | [–0.084,  –0.026] | [–0.065,  –0.025] | [–0.065,  –0.026] | [–0.066,  –0.026] | [–0.066,  –0.026] | [–0.065,  –0.025] | [–0.017, 0.024] | [–0.017, 0.023] | [–0.016,  0.022] | [–0.017,  0.023] | [–0.019,  0.024] |
| Self-empl × 2018 | –0.078 | –0.078 | –0.079 | –0.080 | –0.079 | –0.036 | –0.036 | –0.037 | –0.036 | –0.036 | –0.005 | –0.005 | –0.005 | –0.005 | –0.006 |
|  | [–0.095,  –0.061] | [–0.096,  –0.061] | [–0.096,  –0.061] | [–0.097,  –0.063] | [–0.096,  –0.062] | [–0.062,  –0.010] | [–0.062,  –0.010] | [–0.063,  –0.010] | [–0.063,  –0.010] | [–0.063,  –0.010] | [–0.018,  0.008] | [–0.018,  0.008] | [–0.017,  0.008] | [–0.019,  0.008] | [–0.019,  0.007] |
| Temporary × 2009 | –0.013 | –0.013 | –0.013 | –0.014 | –0.015 | –0.007 | –0.007 | –0.007 | –0.007 | –0.007 | –0.003 | –0.003 | –0.003 | –0.004 | –0.003 |
|  | [–0.024,  –0.002] | [–0.024,  –0.002] | [–0.024,  –0.002] | [–0.025,  –0.003] | [–0.027,  –0.003] | [–0.015,  0.001] | [–0.015,  0.001] | [–0.015,  0.001] | [–0.015,  0.001] | [–0.015,  0.001] | [–0.006,  0.000] | [–0.006,  –0.000] | [–0.006,  –0.000] | [–0.007,  –0.001] | [–0.006,  0.000] |
| Temporary × 2012 | –0.025 | –0.025 | –0.026 | –0.026 | –0.027 | –0.018 | –0.019 | –0.019 | –0.019 | –0.019 | –0.002 | –0.002 | –0.002 | –0.003 | –0.003 |
|  | [–0.037,  –0.013] | [–0.037,  –0.013] | [–0.037,  –0.014] | [–0.038,  –0.015] | [–0.039,  –0.015] | [–0.027,  –0.010] | [–0.027,  –0.010] | [–0.027,  –0.010] | [–0.028,  –0.011] | [–0.028,  –0.011] | [–0.006,  0.002] | [–0.006,  0.001] | [–0.006,  0.001] | [–0.007,  0.001] | [–0.007,  0.001] |
| Temporary × 2015 | –0.032 | –0.033 | –0.033 | –0.034 | –0.033 | –0.016 | –0.017 | –0.017 | –0.018 | –0.018 | –0.006 | –0.006 | –0.006 | –0.006 | –0.007 |
|  | [–0.044,  –0.021] | [–0.044,  –0.021] | [–0.044,  –0.022] | [–0.045,  –0.023] | [–0.043,  –0.022] | [–0.025,  –0.008] | [–0.026,  –0.008] | [–0.026,  –0.008] | [–0.026,  –0.009] | [–0.026,  –0.009] | [–0.009,  –0.003] | [–0.009,  –0.003] | [–0.009,  –0.003] | [–0.009,  –0.003] | [–0.010,  –0.004] |
| Temporary × 2018 | –0.027 | –0.027 | –0.028 | –0.028 | –0.028 | –0.009 | –0.010 | –0.010 | –0.010 | –0.010 | –0.000 | –0.000 | –0.001 | –0.001 | –0.001 |
|  | [–0.038,  –0.016] | [–0.038,  –0.016] | [–0.039,  –0.017] | [–0.039,  –0.017] | [–0.039,  –0.018] | [–0.018, 0.000] | [–0.019,  –0.000] | [–0.019,  –0.000] | [–0.019,  –0.000] | [–0.019,  –0.001] | [–0.004, 0.004] | [–0.004, 0.003] | [–0.004,  0.003] | [–0.005,  0.003] | [–0.005,  0.004] |
| Age, age-squared, education | Yes | Yes | Yes | Yes | Yes | Yes | Yes | Yes | Yes | Yes | Yes | Yes | Yes | Yes | Yes |
| Social Class (ESeG) | No | Yes | Yes | Yes | Yes | No | Yes | Yes | Yes | Yes | No | Yes | Yes | Yes | Yes |
| Net salary | No | No | Yes | No | Yes | No | No | Yes | No | Yes | No | No | Yes | No | Yes |
| Partner’s Class (ESeG) | No | No | No | Yes | Yes | No | No | No | Yes | Yes | No | No | No | Yes | Yes |
| Couple net salary | No | No | No | Yes | Yes | No | No | No | Yes | Yes | No | No | No | Yes | Yes |
| Regional unemployment (squared) | No | No | No | No | Yes | No | No | No | No | Yes | No | No | No | No | Yes |
| Observations | 160230 | 160230 | 160230 | 160230 | 160230 | 258171 | 258171 | 258171 | 258171 | 258171 | 320285 | 320285 | 320285 | 320285 | 320285 |

Source: LFS, Italy 2000–2020

# **Fig. S1** Moderation by education, net of social class (ESeG) and current net salary


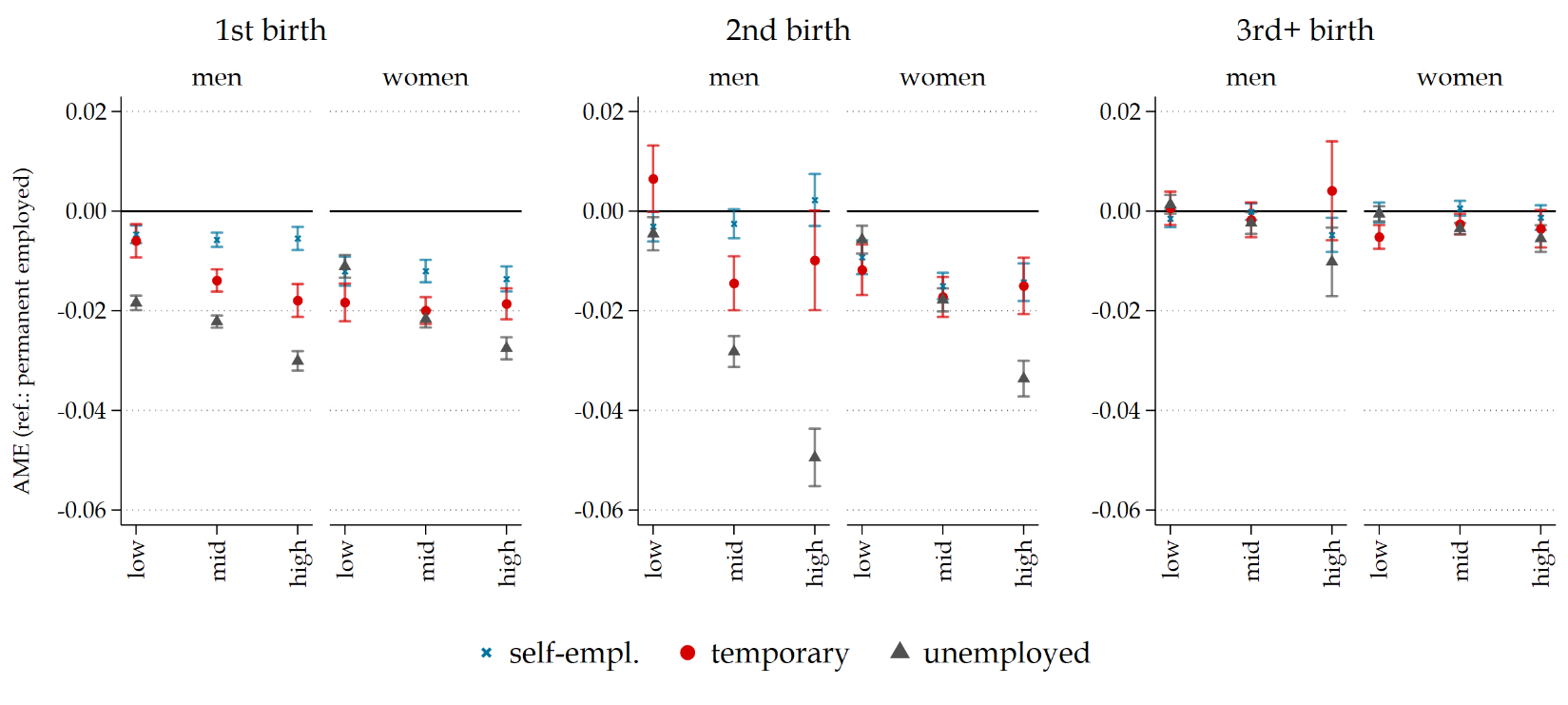


Source: LFS, Italy 2000–2022. Note: Average Marginal Effects (AME) from logistic regression models examining first, second, third child as a function of employment situation (1-year lagged), by sex and educational level, controlling for age, age–squared, period, social class (ESeG), and current net salary deciles, with 95% Confidence Intervals. “Low” refers to below upper secondary, “mid” to upper secondary, and “high” to tertiary level education.

# **Fig. S2** Own and partner’s employment situation, net of partners’ class and current couple’s net salary


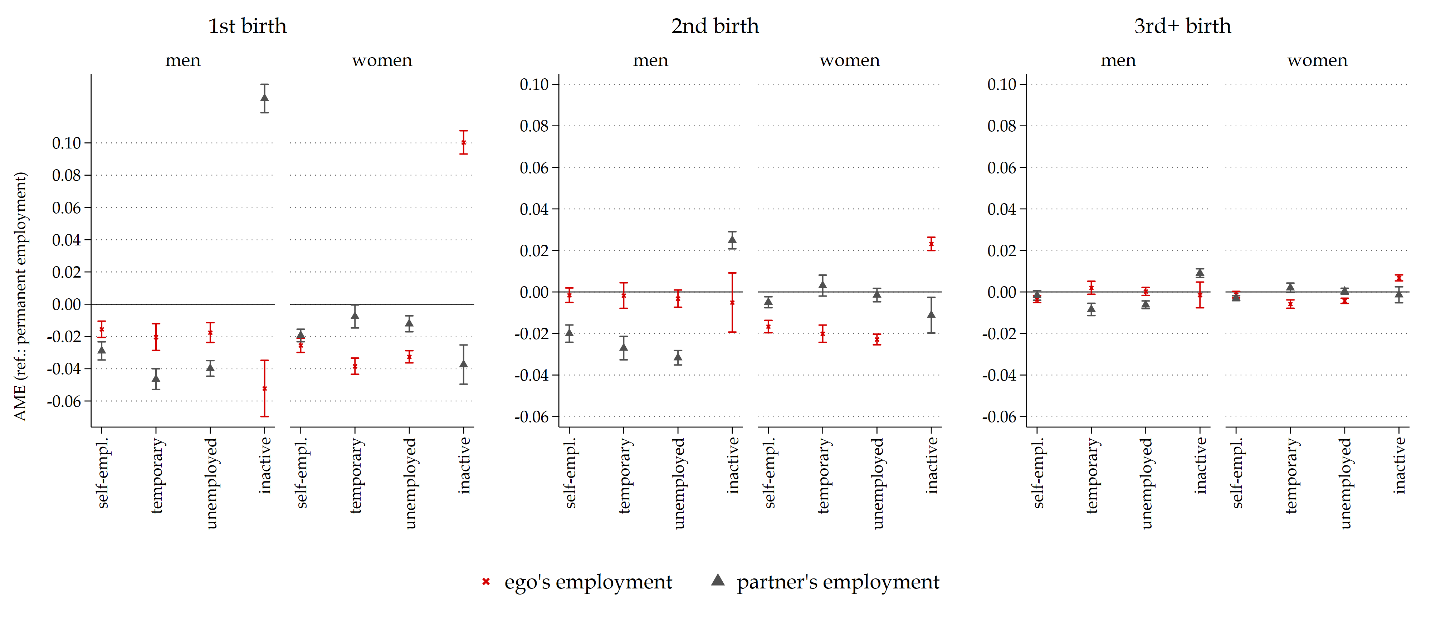


Source: LFS, Italy 2009–2020. Note: Average Marginal Effects (AME) from logistic regression models examining first, second, and higher childbirths as a function of one’s own employment situation (1-year lagged) and partner’s employment situation (1-year lagged), by sex, controlling for age, age-squared, level of education, period, both partners’ social class (ESeG) and current couple’s net salary deciles, with 95% Confidence Intervals.
